# The Tyrosine Kinase Tec Regulates Effector Th17 Differentiation, Pathogenicity, and Plasticity in T-Cell-Driven Intestinal Inflammation

**DOI:** 10.3389/fimmu.2021.750466

**Published:** 2021-12-21

**Authors:** Lisa Sandner, Marlis Alteneder, Ci Zhu, Anastasiya Hladik, Sandra Högler, Ramona Rica, Lars W. Van Greuningen, Omar Sharif, Shinya Sakaguchi, Sylvia Knapp, Lukas Kenner, Michael Trauner, Wilfried Ellmeier, Nicole Boucheron

**Affiliations:** ^1^ Division of Immunobiology, Institute of Immunology, Center for Pathophysiology, Infectiology and Immunology, Medical University of Vienna, Vienna, Austria; ^2^ Division of Gastroenterology and Hepatology, Department of Medicine III, Medical University of Vienna, Vienna, Austria; ^3^ Department of Medicine 1, Research Laboratory of Infection Biology, Medical University of Vienna, Vienna, Austria; ^4^ Unit of Laboratory Animal Pathology, Department for Pathobiology, University of Veterinary Medicine Vienna, Vienna, Austria; ^5^ Department of Medical Biochemistry, Leiden Institute of Chemistry, Leiden, Netherlands; ^6^ Center for Physiology and Pharmacology, Institute for Vascular Biology and Thrombosis Research, Medical University of Vienna, Vienna, Austria; ^7^ Christian Doppler Laboratory for Arginine Metabolism in Rheumatoid Arthritis and Multiple Sclerosis, Vienna, Austria; ^8^ Department of Pathology, Medical University of Vienna, Vienna, Austria; ^9^ Division of Experimental and Translational Pathology, Department of Pathology, Medical University Vienna, Vienna, Austria; ^10^ Center for Biomarker Research in Medicine (CBmed), Graz, Austria; ^11^ Christian Doppler Laboratory for Applied Metabolomics (CDL-AM), Division of Nuclear Medicine, Department of Biomedical Imaging and Image-Guided Therapy, Medical University of Vienna, Vienna, Austria

**Keywords:** Th17 cells, Tec kinases, differentiation, plasticity, colitis

## Abstract

T helper (Th) 17 cells are not only key in controlling infections mediated by extracellular bacteria and fungi but are also triggering autoimmune responses. Th17 cells comprise heterogeneous subsets, some with pathogenic functions. They can cease to secrete their hallmark cytokine IL-17A and even convert to other T helper lineages, a process known as transdifferentiation relying on plasticity. Both pathogenicity and plasticity are tightly linked to IL-23 signaling. Here, we show that the protein tyrosine kinase Tec is highly induced in Th17 cells. Th17 differentiation was enhanced at low interleukin-6 (IL-6) concentrations in absence of Tec, which correlates with increased STAT3 phosphorylation and higher *Il23r* expression. Therefore, we uncovered a function for Tec in the IL-6 sensing *via* STAT3 by CD4^+^ T cells, defining Tec as a fine-tuning negative regulator of Th17 differentiation. Subsequently, by using the IL-17A fate mapping mouse combined with *in vivo* adoptive transfer models, we demonstrated that Tec not only restrained effector Th17 differentiation but also pathogenicity and plasticity in a T-cell intrinsic manner. Our data further suggest that Tec regulates inflammatory Th17-driven immune responses directly impacting disease severity in a T-cell-driven colitis model. Notably, consistent with the *in vitro* findings, elevated levels of the IL-23 receptor (IL-23R) were observed on intestinal pre- and postconversion Th17 cells isolated from diseased *Tec^−/−^* mice subjected to adoptive transfer colitis, highlighting a fundamental role of Tec in restraining IL-23R expression, likely *via* the IL-6-STAT3 signaling axis. Taken together, these findings identify Tec as a negative regulator of Th17 differentiation, pathogenicity, and plasticity, contributing to the mechanisms which help T cells to orchestrate optimal immune protection and to restrain immunopathology.

## Introduction

CD4^+^ T helper (Th) cells are divided into specialized subsets. Among these subsets, Th17 cells secrete the hallmark cytokine interleukin-17A (IL-17) and mediate host defense against pathogenic extracellular bacteria and fungi (1–3). Under physiologic conditions, Th17 cells reside at mucosal sites such as the intestinal lamina propria and Peyer’s patches, where they function as a first line of defense (1, 4–6). However, improper Th17 differentiation contributes to the development of autoimmune diseases (7). Therefore, it is of utmost importance to characterize the signaling network within CD4^+^ T cells that regulates Th17-specific transcriptional circuits as this has profound clinical implications for a broad range of diseases (8, 9).

Activation of the T-cell receptor (TCR) of naïve CD4^+^ T cells in presence of Transforming Growth Factor β1 (TGF-β1) and IL-6 leads to the induction of a complex Th17-specific transcriptional program which includes signal transducer and activator of transcription 3 (STAT3), retinoic‐acid‐receptor‐related orphan nuclear receptor α (RORα) and the lineage‐specific transcription factor RORγt (10–15). TGF-β1 signaling *via* Smad dependent and independent pathways together with IL-6 signaling *via* activation of Jak kinases and the Jak-mediated phosphorylation of STAT3 proteins (16) lead to efficient induction of *Il17a* and *Rorc* gene expression. In addition, IL-6 signaling induces upregulation of the Th17 driving cytokine IL-21, and the IL-1 (IL-1R) and IL-23 (IL-23R) receptors (16, 17). IL-1 and IL-23 were shown to support Th17 differentiation, expansion, and maintenance (17–19).

Importantly, IL-1 and IL-23 were also shown to convert Th17 cells from a so-called protective noninflammatory subset to a rather pathogenic one, which drives inflammation in various autoimmune conditions (9, 20). Therefore, Th17 cells can be subdivided into further subsets with specific functions and cytokine expression profiles. This diversity is further accentuated by a high degree of plasticity and instability as Th17 cells were shown to express cytokines from other Th lineages (like the Th1-specific IFN-γ) and to loose expression of their lineage-specific Il-17, converting to Th1, T regulatory 1 (Tr1), or T follicular helper cells (Tfh) (21–23). The complex transcriptional network leading to Th17-cell differentiation has been investigated in detail *via* systems biology approaches (24, 25), which have provided new insights into the regulation of early and late transcription factors recruitment, chromatin remodeling and plasticity of Th17 cells.

Additionally, significant information has been gained concerning signaling events controlling Th17 differentiation (26). In particular, tyrosine kinase expressed in hepatocellular carcinoma (Tec) family members were shown to regulate T helper cell differentiation (27) and in particular Th17 cell differentiation (28–31). Three members of the Tec kinase family are expressed in T cells: Tec, the founder member of this kinase family, inducible interleukin-2-inducible T-cell kinase (Itk), and resting lymphocyte kinase (Rlk). Itk was shown to direct the lineage choice between FOXP3^+^ regulatory T cells (Treg) and Th17 cells (29), linking TCR and cytokine signaling and acting *via* the mTOR signaling pathway. We showed that Tec negatively regulates a memory Th17 subset *in vivo* (28). Others showed that Tec phosphorylates cMaf, thereby enhancing its recruitment to the *Il21* promoter, promoting IL-21 expression in Th17 cells (31). However, further investigations are required to better understand the mechanisms by which Tec controls Th17 differentiation. Deciphering the position of Tec in the early signaling events in CD4^+^ T cells affecting Th17 differentiation and how Tec shapes Th17 differentiation *in vivo* during an immune response and in a disease setting remains to be clarified.

Here, using naïve CD4^+^ T cells from Tec-deficient mice, we demonstrate that Tec dampens Th17 cell differentiation *in vitro* by acting as a negative regulator of the IL-6 signaling pathway. Mechanistically, this dampening function was associated with a decrease in STAT3 phosphorylation at low IL-6 concentrations, thereby regulating the activity of a transcription factor critical for Th17 differentiation. In addition, we show that Tec was highly induced in Th17 cells. Furthermore, Th17 differentiation and plasticity towards Th1-like cells was increased *in vivo* in a T-cell intrinsic way, and Tec-deficient naïve CD4^+^ T cells differentiated more into Th1/exTh17 cells in a colitis model, leading to a stronger disease outcome. Moreover, Tec regulated the expression of *Il23r in vitro* at low IL-6 concentration during Th17 differentiation, and the levels of IL-23R on pre- and postconversion Th17 cells *in vivo* during colitis, implying that by restraining IL-6 signaling, Tec also restrains STAT3 phosphorylation, IL-23 receptor levels, and therefore full differentiation and plasticity of Th17 cells. Collectively, our data identify Tec as a fine-tuning regulator of IL-6 signaling *via* STAT3 in Th17 differentiation *in vitro* and of Th17 differentiation and plasticity *in vivo*.

## Materials and Methods

### Mice

Mice deficient for Tec (*Tec^−/−^* mice) were described previously (32). OT-II TCR transgenic mice were kindly provided by Dr. Stoiber-Sakaguchi (Medical University of Vienna). C57BL/6 *Rag1^−/−^*, C57BL/6 *Rag2^−/−^*, and Rosa26^flox^STOP^flox^ eYFP mice were purchased from The Jackson Laboratories. IL-17A^CRE^ mice were kindly provided by B. Stockinger (21).

The IL-17 fate mapping mice resulted from the breeding of WT R26^YFP^ or *Tec^−/−^* R26^YFP^ mice with WT IL-17A^CRE^ or *Tec^−/−^* IL-17A^CRE^ mice, respectively. All mice were bred and maintained in the preclinical research facility of the Medical University of Vienna, and animal experiments were done according to protocols approved by the Federal Ministry for Education, Science and Art (GZ:BMWF-66.009/0105-WF/II/3b/2014, BMBWF-66.009/0039-V/3b/2019). Animal experiments were performed under national laws in agreement with guidelines of the Federation of European Laboratory Animal Science Associations (FELASA), which correspond to the National Center for the Replacement, Refinement, and Reduction of Animals in Research (ARRIVE) guidelines.

### Flow Cytometric Analysis, Antibodies, and Reagents

The following antibodies and reagents were used in our study: from BD Biosciences (Franklin Lakes, NJ, USA): IL-17A (TC11-18H10.1), IL-10 (JES5-16E3), pSTAT1 (4a), pSTAT3 (4/P-STAT3), pSTAT4 (38/p-Stat4), and pSTAT5 (47); from Thermo Fisher (Waltham, MA, USA): CD4 (clone RM4-5), CD8a (53-6,7), CD45.1 (A20), CD45.2 (104), Foxp3 (FJK-16s), IL-22 (IL22JOP), and fixable viability dye eFluor™ 506; and from BioLegend (San Diego, CA, USA): CD16/CD32 (2.4G2), CD44 (IM7), CD19 (6D5), CD45.2 (104), CD62L (MEL-14), CD45RB (MB4B4), IFN-γ (XMG1.2), IL-23R (12B2B64), TCR-β (H57-597), and IL-6Rα (D7715A7). The clone number is indicated in brackets.

### Differentiation of CD4^+^ T Cells and Cytokine Measurements

CD4^+^ T cells were isolated from spleen and lymph nodes of 8-week-old mice as previously described (28). Sorted naive (CD44^low^CD62L^+^) CD4^+^ T cells were stimulated (day 0) with plate-bound anti-CD3ϵ (1 µg/ml) and anti-CD28 (3 µg/ml) on 96-well plates (2 × 10^5^ cells/well) in 200 µl T-cell medium (RPMI GlutaMAX-I supplemented with 10% FCS, antibiotics, and 2-ME; all from Invitrogen, Waltham, MA, USA) under various cytokine conditions as indicated in the figure legend. For Th17 conditions, IL-6 (20 ng/ml, or as indicated in the figure legend) and TGF-β1 (1 ng/ml, or as indicated in the figure legend) were used. Cells were cultured for 72 h or as indicated in the figure legend. Where indicated, IL-1β (20 ng/ml, BioLegend) and IL-23 (20 ng/ml, R&D, Minneapolis, MN, USA) were added to the differentiation medium. Cells were analyzed after 60 h by flow cytometry on a LSRII Fortessa (BD Biosciences).

### Intracellular Cytokine, Transcription Factor, and pSTAT Stainings

T cells were cultured under various conditions as described above. For intracellular cytokine detection, cells were stimulated in 96-well plates for 3 h with PMA (50 ng/ml) and ionomycin (500 ng/ml) (Sigma-Aldrich, St. Louis, MO, USA) in the presence of GolgiStop (BD Biosciences). After harvesting, T cells were surface stained with appropriate antibodies. Subsequently, cells were fixed for 10 min with Cytofix Fixation Buffer (BD Biosciences), permeabilized 15 minutes with Perm/Wash Buffer (BD Biosciences), and stained for intracellular proteins according to the manufacturer’s protocol. Intracellular phosphorylated STAT levels were assessed using BD Phosflow protocol.

### cDNA Synthesis and Quantitative Real-Time PCR

Cells were harvested, and total RNA was isolated with Qiagen RNA isolation kit according the manufacturer’s instructions. RNA was reversely transcribed using the iScript cDNA synthesis kit (Bio-Rad, Hercules, CA, USA). Quantitative real-time PCR (qRTPCR) analysis was performed with the SuperScript III qPCR MasterMix (Invitrogen) on the CFX 96 Real-Time PCR detection system (Bio-Rad). The list of primers used can be found in the **Supplementary Table**.

### Cytometric Bead Arrays and ELISAs for Cytokine Measurements

Mouse IL-17A and IFN-γ ELISA MAX Deluxe sets (BioLegend) and Mouse IL-21 DuoSet Elisa (R&D) were used to measure IL-17A, IFN-γ, and IL-21 from the culture supernatant of Th17 cultures from CD4^+^ T cells isolated from WT or Tec-deficient mice.

### Immunoblot Analysis

T cells (0.5 × 10^6^) were lysed in (50 µl) Carin lysis buffer (20 mM Tris-HCl [pH 8.0], 138 mM NaCl, 10 mM EDTA, 100 mM NaF, 1% Nonidet P-40, 10% glycerol, 2 mM Na orthovanadate) supplemented with complete protease inhibitors (Roche, Basel, Switzerland). Proteins were separated on 10% SDS-polyacrylamide gels and blotted onto PVDF membranes according to standard protocols. The following primary antibodies were used: anti-actin (AC-74; Sigma-Aldrich, St. Louis, MO, USA) and rabbit anti-Tec. Secondary antibody was a peroxidase-conjugated AffiniPure goat anti-rabbit IgG (H+L) (Jackson ImmunoResearch Laboratories, West Grove, PA, USA). Signals were detected using ECL (SuperSignal West Dura Extended Duration Substrate from ThermoScientific).

### T-Cell Transfer Experiment and Immunization

Recipient mice (CD45.1^+^) received 5 × 10^5^ CD45.2^+^ *Tec* ^−/−^ or *Tec* ^+/+^ OTII IL-17A^cre^ R26^YFP^ CD4^+^ T cells intravenously 1 day before immunization with 100 μg OVA(323–339) in CFA subcutaneously in the flank. Draining inguinal lymph nodes were isolated, and phenotype of OTII^+^ cells was assessed by flow cytometry, gating on live CD4^+^CD45.2^+^ cells.

### Colitis and Isolation of Lymphocytes From Small Intestine, Colon, and IEL/LPL From Small Intestine

CD4^+^CD45RB^hi^CD25^−^CD45.2^+^ T cells were purified from CD4^+^ T cells isolated from spleens and LNs of *Tec^−/−^* and control mice, and intraperitoneally injected into *Rag1* ^−/−^ CD45.1^+^ mice or *Rag2^−/−^* CD45.2^+^ mice (4 × 10^5^ cells per mouse) as indicated in the figures. After 3 or 7 weeks, as indicated in the figures, mice were sacrificed and intestines removed and placed in ice-cold PBS. The colon length was measured, and the tissue was kept for histology or used for cell isolation. After removal of residual mesenteric fat tissue, the small intestine or colon was opened longitudinally. The intestine was then thoroughly washed in ice-cold PBS and cut into 1.5 cm pieces. The pieces were washed twice with 10 mM Hepes, 10% fetal bovin serum in Hanks’ balanced salt solution (HBSS) to remove excess mucus and incubated twice in 5 ml of 5 mM EDTA in HBSS for 15–20 min at 37°C with shaking. After each incubation, the epithelial cell layer, containing the intraepithelial lymphocytes (IELs), was removed by intensive vortexing and passing through a 100-μm cell strainer, and new EDTA solution was added. After the second EDTA incubation, the pieces were washed in HBSS, cut in small pieces using scissors, and placed in 5 ml digestion solution containing 4% fetal calf serum and 0.5 mg/ml each of collagenase D (Roche) and DNase I (Sigma). For isolation of lymphocytes from small intestine or colon 3 weeks after transfer, cells were not incubated with EDTA for IEL isolation and processed directly for digestion. Digestion was performed by incubating the pieces at 37°C for 20 min with shaking. After the initial 20 min, the solution was vortexed intensely and passed through a 40-μm cell strainer. The pieces were collected and placed into fresh digestion solution, and the procedure was repeated a total of three times. Supernatants from all three digestions (or from the EDTA treatment for IEL isolation) from a single small intestine were combined, washed once in cold FACS buffer, resuspended in 10 ml of the 40% fraction of a 40:80 Percoll gradient, and overlaid on 5 ml of the 80% fraction in a 15-ml Falcon tube. Percoll gradient separation was performed by centrifugation for 20 min at 2,500 rpm at room temperature. Lamina propria lymphocytes (LPLs) were collected at the interphase of the Percoll gradient, washed once, and resuspended in FACS buffer or T-cell medium. The cells were used immediately for experiments.

### Histology

Colon was fixed in 4% buffered formalin and embedded in paraffin. Blocks were cut at 2 µm thickness and stained with hematoxylin and eosin (HE). A pathologist blinded to treatment groups evaluated slides in a semiquantitative manner. Extent and severity of the inflammatory cell infiltrate (1-5), epithelial changes (1-5), and lesions of the mucosal architecture (1-5) were graded according to a scoring scheme for colonic inflammation mediated by disturbed immune cell homeostasis (33). Furthermore, crypt length from surface of the epithelium to the lamina muscularis mucosae was measured with an Olympus BX53 microscope and attached Olympus DP26 camera in three locations of each colon perpendicular to the serosal surface. The same microscope and camera were used for acquisition of representative images. White balance and assembly of images were done using Adobe Photoshop 2021. The total histopathological score was calculated by adding the scores for each of the parameters mentioned above as described in (34).

### RNA-Sequencing—Data Origin and Processing

RNA-sequencing data were downloaded as reads per kilobase of transcript, per million mapped reads (RPKM) from NCBI GEO datasets (GSE16452, GSE163894, GSE168288, (35)), and Tec expression was normalized to Hprt.

### Statistical Analysis

All data are expressed as mean with SEM. Statistical analysis was performed by using a nonpaired Student’s *t*-test or by one-way ANOVA corrected with Tukey *post-hoc* test as indicated in the figure legend. For multiple comparison testing, two-way ANOVA using a Sidak’s multiple comparisons test was applied as indicated in the figure legend. The *p*-values were defined as follows: ^*^ *p* < 0.05; ^**^ *p* < 0.01; ^***^ *p* < 0.001; n.s., not significant.

## Results

### Tec Isoform a Is Predominantly Upregulated in Th17 Cells

**Figure 1:**
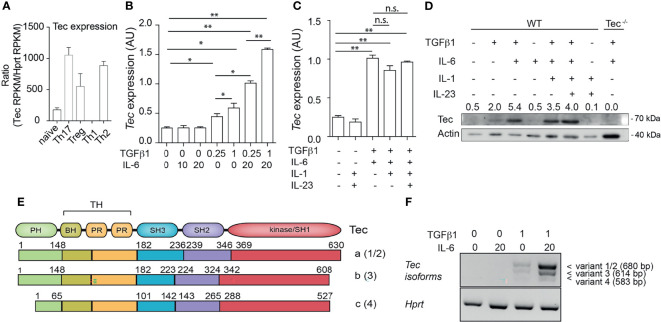
Up-regulation of Tec isoform a under Th17 differentiation conditions. **(A)** Expression level of *Tec* among T helper lineages. Data were retrieved from NCBI public data sets and are expressed as Reads Per Kilobase of transcript, per Million mapped reads (RPKM). Expression of *Tec* was normalized to *Hprt* expression. **(B)** Expression of *Tec* was determined by RT-qPCR in naïve CD4^+^ T cells stimulated with immobilized anti-CD3 and anti-CD28 for 3 days under various TGFβ1 and IL-6 concentrations, as indicated in the figure below the graph. Data are represented as mean ± SEM as summary from 2 independent experiments with 3 replicates each. **(C)** Expression of *Tec* was determined by RT-qPCR in naïve CD4^+^ T cells stimulated as in **(A)** under various cytokine conditions as indicated in the figure. Data are represented as mean ± SEM as summary from 2 independent experiments with 3 replicates each. **(D)** Tec protein expression was monitored by SDS-PAGE in naïve CD4^+^ T cells stimulated as in **(B)** under the indicated cytokine conditions. Numbers above the blot indicate the band intensity quantification normalized to actin. As a control naïve CD4^+^ T cells from *Tec-/-* mice were added. One representative experiment out of 2 is shown. **(E)** Scheme depicting the protein structure and isoforms of Tec. Number between brackets indicate the splice variant origin. Splice variants 1 and 2 have different regulatory regions upstream of the initiation codon and both result in isoform a, splice variant 3 results in isoform b and splice variant 4 in isoform c. **(F)** Tec isoforms were determined in naïve CD4^+^ T cells stimulated as in **(A)** under various cytokine conditions as indicated in the figure by PCR using isoform specific primers. *Hprt* was used as house-keeping gene. One representative experiment out of two is shown. P values were calculated by one-way ANOVA corrected with Tukey post-hoc test. *P < 0.05, **P < 0.01. n.s., non significant. AU, Arbitrary Units. PH, pleckstrin homology domain. TH, Tec homology. BH, Btk homology. PR, proline rich. SH, SRC homology. Aa, amino acid.

Tec kinases were shown to have a T helper-cell subtype-specific expression pattern (28, 36). We revisited *Tec* expression profiles among T helper subsets by reanalyzing published RNA-sequencing data (35) (see *Result* section for complete datasets used) (**Figure 1A**). *Tec* was expressed at low levels in naïve and Th1 cells, exhibiting an intermediate expression level in Treg cells and highest expression in Th2 and Th17 cells (**Figure 1A**). To investigate how *Tec* expression is regulated in Th17 cells, we isolated naïve CD4^+^ T cells from WT mice and cultured them with TGF-β1 and/or IL-6 (**Figure 1B**). Different concentrations of either TGF-β1 or IL-6 were used in order to test a dose dependency. The cells were then further processed for assessment of *Tec* mRNA levels. Addition of TGF-β1 alone but not IL-6 led to a mild induction of *Tec* in a dose-dependent manner. However, the combination of TGF-β1 and IL-6 resulted in the highest induction of *Tec*, indicating a synergistic activity of TGF-β1 and IL-6 signaling pathways in inducing *Tec* expression. Other Th17 driving cytokines like IL-1 or IL-23 did not further enhance *Tec* induction (**Figure 1C**). We confirmed the mRNA expression data on a protein level (**Figure 1D**). Naïve CD4^+^ T cells cultured under Th17 driving conditions from *Tec^−/−^* mice were used as negative control. An analysis of the *Tec* locus on the NCBI database indicates several transcript variants (1 and 2, 3, and 4) resulting in 3 Tec protein isoforms (named a, b, and c, respectively) (**Figure 1E**). In Th17 cells, we detected isoform a, the dominant form in immune cells (37), as the major isoform expressed (**Figure 1F**). Taken together, our data show that in Th17 cells, Tec isoform a is predominantly induced *via* synergistic activity of IL-6 and TGF-β1 signaling.

### Tec Dampens IL-6 Signaling During Th17 Differentiation *via* STAT3

**Figure 2:**
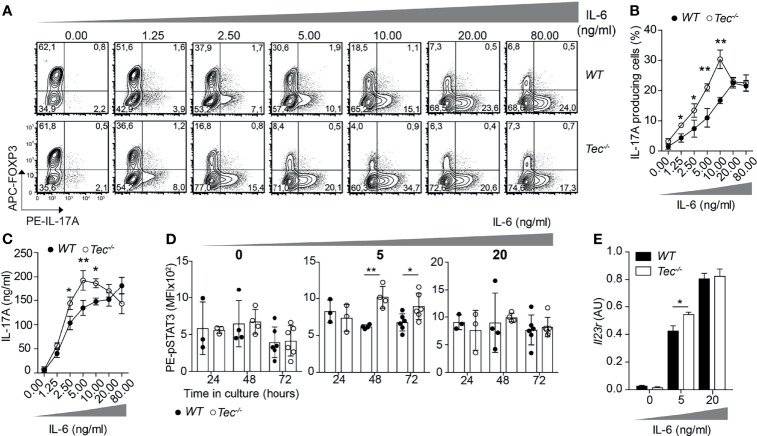
Tec impacts on IL-6 sensing during Th17 differentiation. **(A)** Contour plots show IL-17A and/or Foxp3 positive cells from naïve CD4^+^ T cells from WT and *Tec-/-* mice cultured 3 days with anti-CD3, anti-CD28, 1ng/ml TGFβ1 and increasing concentrations of IL-6 as indicated on the figure. One representative experiment out of 3 is shown. **(B)** Quantification of **(A)**. **(C)** IL-17A secretion was measured from supernatants of cells cultured as in **(A)**. **(D)** Analysis by flow cytometry of STAT3 phosphorylation of naïve WT or *Tec-/-* CD4^+^ T cells cultured with TGFβ1 without or with 5 or 20 ng/ml IL-6 for 24, 48 or 72 hours. **(E)** Il23r expression on naïve CD4^+^ T cells from WT and *Tec-/-* mice cultured as in **(A)** with the indicated IL-6 concentrations and constant 1 ng/ml TGFβ1 concentration. A summary of two experiments with 3 replicates each is shown. P values were calculated by two-way ANOVA using a Sidak’s multiple comparisons test **(C–F)**. *P < 0.05, **P < 0.01.

As Tec is highly induced under Th17 priming conditions, we investigated how TGF-β1 or IL-6 signaling impacts on the frequency of IL-17A-positive cells and IL-17A secretion during activation of WT and *Tec^−/−^* naïve CD4^+^ T cells (**Figure 2**; **Supplementary Figure S1**). Therefore, we activated naïve CD4^+^ T cells from WT or *Tec^−/−^* mice with plate-bound CD3 and CD28 in the presence of a fixed concentration of IL-6 (20 ng/ml) and increasing concentrations of TGF-β1 (**Supplementary Figure S1**). As expected, Th17 differentiation of both WT and *Tec^−/−^* naïve CD4^+^ T cells increased with higher TGF-β1 concentrations until reaching a plateau and even decreasing at higher concentrations. The sensing of various TGF-β1 concentrations by naïve *Tec^−/−^* CD4^+^ cells was comparable with the sensing of naïve WT CD4^+^ T cells. We also activated naïve CD4^+^ T cells from WT or *Tec^−/−^* mice in the presence of a fixed concentration of TGF-β1 (1 ng/ml) and increasing concentrations of IL-6 (**Figures 2A–C**). Again, Th17 differentiation of both WT and *Tec^−/−^* naïve CD4^+^ T cells increased with higher IL-6 concentrations. Notably, *Tec^−/−^* Th17 cells exhibited higher percentages of IL-17A-positive cells and higher IL-17A secretion at IL-6 concentrations below 20 ng/ml (further defined as low IL-6 concentrations) compared with WT Th17 cells implying a stronger IL-6 sensing in the absence of Tec (**Figures 2A–C**). In agreement with the IL-17A secretion data, STAT3 phosphorylation was enhanced in cells cultured for 48 or 72 h with TGF-β1 and low IL-6 concentration in the absence of Tec, equaling out with WT levels at higher IL-6 concentration (**Figure 2D**). Of note, STAT5 phosphorylation in contrast to STAT1 and STAT4 phosphorylation was enhanced in Tec-deficient cells cultured for 48 h with low IL-6 concentration, indicating modulation of other STAT molecules by Tec (**Supplementary Figure S2**). To assess whether Tec directly regulates IL-6R signaling independently of T-cell receptor activation, WT and *Tec^−/−^* naïve CD4^+^ T cells were activated with anti-CD3 and anti-CD28 in the presence of TGF-β1 and IL-6 for 48 h, rested in PBS with 2% FCS, and activated for 15 min with 5 or 20 ng/ml IL-6 (**Supplementary Figure S3A**). A kinetic study of Tec expression revealed that Tec protein is clearly detectable at 48 h (**Supplementary Figure S3B**). STAT3 phosphorylation was enhanced in the absence of Tec with 5 ng/ml IL-6 but not 20 ng/ml IL-6, showing that Tec directly regulates IL-6R signaling at low IL-6 concentration (**Supplementary Figure S3C**). Furthermore, the effects of Tec were specific for the Th17 lineage as Foxp3 and IFN-γ expression in *Tec^−/−^* Th17 cells generated with increasing IL-6 concentrations was similar to WT Th17 cells (**Supplementary Figures S4A, B**). IL-21 secretion which is also dependent on STAT3 activity (38) was increased at 2.5 and 5 ng/ml IL-6 in Tec-deficient conditions (**Supplementary Figure S4B**). As a result of a stronger IL-6 signaling, the expression of *Il23r* was enhanced in *Tec^−/−^* Th17 cells at low IL-6 concentration as compared with their WT counterparts (**Figure 2E**). Of note, the IL-6 receptor levels were similar between WT and *Tec^−/−^* naïve CD4^+^ T cells, excluding a stronger IL-6 response due to higher receptor expression as a consequence of T-cell developmental alterations (**Supplementary Figure S5**). Taken together, our data indicate that Tec dampens Th17 differentiation at low IL-6 concentrations *via* inhibition of STAT3 phosphorylation, and thereby restrains IL-21 secretion and *Il23r* expression.

### Tec Regulates Th17 Effector Differentiation *In Vivo* in a T-Cell-Intrinsic Way

**Figure 3:**
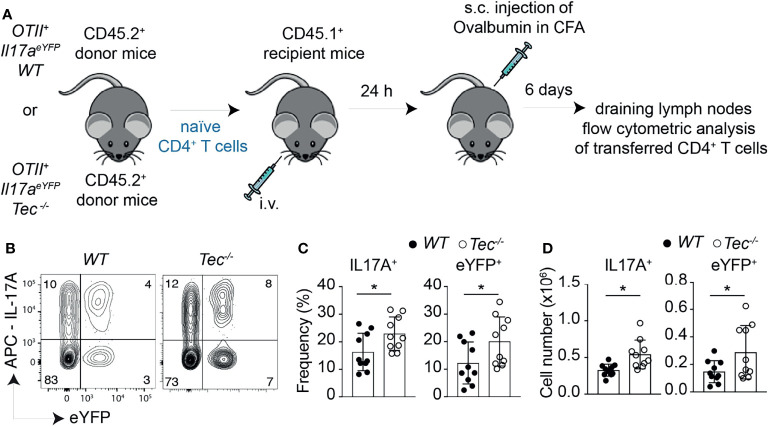
Tec dampens Th17 responses *in vivo*. **(A)** Experimental scheme. **(B)** Dot plots represent IL-17A versus eYFP positive cells isolated from the inguinal lymph nodes after transfer and immunization as depicted in **(A)**. **(C)** Quantification of **(A) (D)** Summary of total cells. The summary of three independent experiments is shown with at least three mice per group. *P* values were calculated by an unpaired two-tailed t test. *P < 0.05.

To study whether the enhanced *in vitro* Th17 differentiation in the absence of Tec can also be observed *in vivo*, we took advantage of an adoptive T-cell transfer model which allows to monitor Th17 differentiation *in vivo* in an antigen-specific way (39) (**Figure 3A**). Th17 cells were shown to either retain IL-17A expression and induce expression of cytokines of other lineages or even switch to another T helper-type-like Th1, Tr1 or Tfh, a feature also described as “plasticity” (11, 21–23, 40, 41). We combined therefore this adoptive transfer model with the IL-17A fate mapping mouse (21) to ensure that we also monitor Th17 cells that switched to another T helper lineage and stopped producing IL-17A, and to further evaluate Th17 *in vivo* plasticity. We transferred a low number of naïve WT or *Tec^−/−^* OT-II IL-17A^cre^ R26^YFP^ CD45.2^+^CD4^+^ T cells (which are specific for ovalbumin, and allowed IL-17A fate mapping) into CD45.1^+^C57BL/6-recipient mice which were subcutaneously immunized 24 h later with ovalbumin in complete Freund’s adjuvant (CFA). Six days later, draining lymph nodes were investigated for IL-17A^+^ cells and eYFP^+^ CD45.2^+^CD4^+^ T cells (for a gating strategy, see **Supplementary Figure S6**). Transferred *Tec^−/−^* CD4^+^ T cells displayed enhanced Th17 differentiation compared with their WT counterpart, as revealed by the expression of IL-17A after PMA/ionomycin *ex vivo* restimulation of draining lymph node cells (**Figures 3B–D**). It was shown that during *in vitro* and *in vivo* Th17 differentiation, eYFP-positive cells correlate partially with IL-17A expression (21). This difference (IL-17A^+^YFP^–^ cells) was explained by a threshold Cre protein expression level required to faithfully mark Th17 cells which have acquired full effector functions. Therefore eYFP^+^ CD4^+^ T cells represent a subset of more mature effector Th17 cells (21). We observed more eYFP^+^ CD45.2^+^CD4^+^ T cells from *Tec^−/−^* mice compared with those from WT mice, indicating that Tec deficiency results in an enhanced fraction of mature effector Th17 cells (**Figures 3B–D**).

### *Tec^−/−^* CD4^+^ T Cells Differentiate More Towards Inflammatory IFN-γ^+^Th17 Cells *In Vivo*

**Figure 4:**
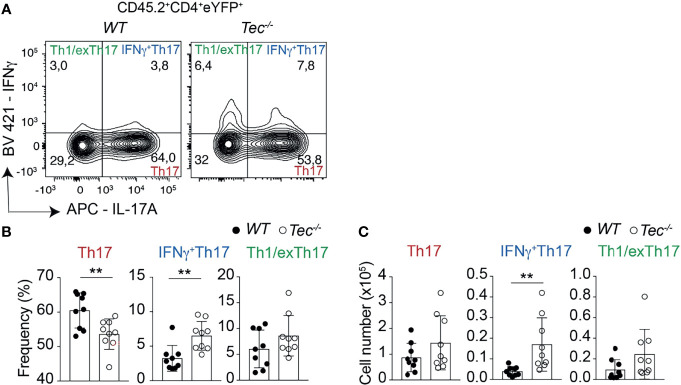
Tec regulates IFNγ^+^Th17 cells. **(A)** Contour plots represent the frequency of IL-17A and IFNγ positive cells isolated from the inguinal lymph nodes after transfer and immunization as depicted in Figure 3 and gated on eYFP+ transferred CD4+ T cells. **(B)** Summary of **(A)**. **(C)** Summary of total cell numbers of IL-17A and IFNγ single and double producing cells resulting from the analysis performed in Figure 4A. The summary of three independent experiments is shown with three mice per group. *P* values were calculated by an unpaired Two-tailed t test. **P < 0.01.

Independent of the genotype, immunization led to a robust induction of Th17 and Th1 cells, but only to a marginal IL-10 production by CD4^+^ T cells, indicating an ongoing inflammatory immune reaction which is still not resolving (**Supplementary Figures S7A–C**) (23). To understand if, in addition to an enhanced Th17 effector differentiation, *Tec^−/−^* CD4^+^ T cells show an altered plasticity and/or Th17 subset distribution *in vivo*, we analyzed the proportion of IL-17 and IFN-γ single- and double-positive CD4^+^ T cells gated on eYFP^+^ cells among transferred CD45.2^+^CD4^+^ T cells in the draining lymph nodes of CD45.1^+^ immunized recipient animals (**Figure 4**; **Supplementary Figure S8** for the gating strategy). This allows the analysis of Th17 cells which produce only IL-17A, IFN-γ^+^Th17 cells which secrete both IL-17A and IFN-γ and Th1/exTh17 cells which secrete only IFN-γ. Upon transfer, *Tec^−/−^* CD4^+^ T cells differentiated more towards IFN-γ^+^Th17 cells (**Figure 4**), suggesting a higher degree of pathogenicity in the absence of Tec, as these cells were described to have a higher pathogenic potential (12). Taken together, our results indicate that Tec regulates the differentiation of Th17 cells and in particular IFN-γ^+^Th17 cells during immunization.

### Loss of Tec in CD4^+^ T Cells Leads to an Enhanced Th17 Differentiation During Onset of T-Cell-Driven Colitis

**Figure 5:**
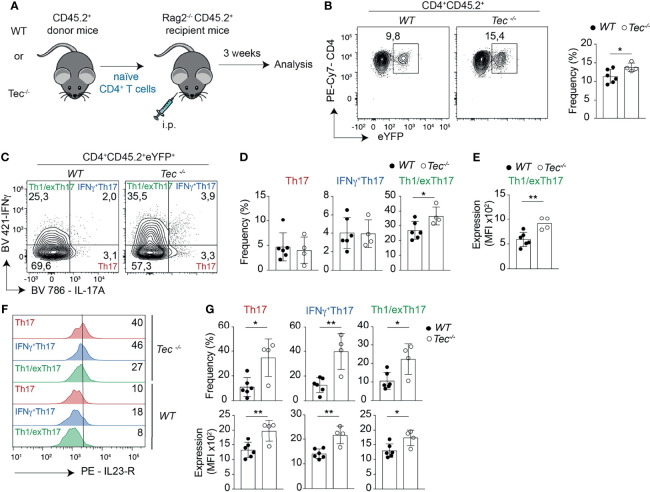
Tec enhances Th17 differentiation and plasticity in the small intestine during onset of adoptive transfer colitis. **(A)** Experimental scheme. **(B)** Contour plots represent the frequency of eYFP^+^ CD4^+^ T cells from small intestine after gating on transferred CD45.2^+^ cells. The summary is represented alongside. **(C)** Contour plots represent the frequency of IL-17A and IFNγ positive intestinal cells gated on eYFP^+^ CD45.2^+^ CD4^+^ T cells from WT and *Tec-/-* diseased mice. **(D)** Summary of (C). **(E)** The scatter plot represents the expression of level of IFNγ; among CD45.2^+^CD4^+^eYFP^+^IFNγ^+^IL-17A^-^ T cells gated as in **(C)**. **(F)** Flow cytometry analysis showing IL-23 receptor expression on the indicated cell type and genotype. The numbers represent the frequency of positive cells. **(G)** Summary of the frequencies (upper scatter plots) and expression levels (lower scatter plots) of the IL-23 receptor from cells represented in **(F)**. **(C–E, G)** represent two independent biological replicates with 3 animals per replicate for the WT group and 2 animals per replicate for the *Tec-/-* group P values were calculated by an unpaired Two-tailed t test. *P < 0.05, **P < 0.01.

Th1 and Th17 cells and especially IFN-γ^+^Th17 cells and Th1/exTh17 cells were shown to play an important role in inflammatory bowel disease (IBD) (42, 43). To understand whether in the absence of Tec an enhanced mucosal Th17 cell differentiation occurs during gut-related inflammatory conditions and disease, and even contributes to the disease severity, we took advantage of the T-cell-driven colitis model (44, 45). In this model, naïve CD4^+^ T cells are adoptively transferred into *Rag2^−/−^* mice deficient for T and B cells (**Figure 5A**). These transferred naïve CD4^+^ T cells develop into Th1 and Th17 effector cells by exposure to gut microbiota antigens and cause severe colitis which recapitulates major aspects of human IBD (46). We therefore transferred naïve WT or *Tec^−/−^* naïve CD4^+^ T cells from IL-17A fate mapping mice into *Rag2^−/−^* mice and analyzed the small intestine and colon for appearance of preconversion (Th17) and postconversion (exTh17) effector cells 3 weeks after transfer by monitoring eYFP^+^ cells (**Figure 5A**; **Supplementary Figure S9A**), a time frame which corresponds to the onset of colitis disease (47), as exemplified by similar colon lengths between control and treated groups (**Supplementary Figure S9B**). Despite similar disease severity, we observed an increase in eYFP^+^ cells in small intestine and colon of mice transferred with *Tec^−/−^* naïve CD4^+^ T cells compared with those that received WT cells (**Figure 5B**; **Supplementary Figure S9C**). Therefore, more Tec-deficient naïve CD4^+^ T cells underwent Th17 differentiation before manifestation of strong disease phenotypes.

### Tec Regulates Plasticity of Th17 Cells During Onset of Colitis

Th17 cells have a high degree of functional heterogeneity and plasticity (41), in particular in the gut (48, 49), where pathogenic IFN-γ producing Th17 cells and anti-inflammatory IL-10 producing Th17 cells were described. Therefore, we analyzed the production of IL-17A versus IFN-γ and IL-17A versus IL-10 by eYFP^+^ CD4^+^ T cells (**Figure 5C**; **Supplementary Figures S9D**, **10**) to identify IFN-γ^+^ Th17, Th1/exTh17, IL-10^+^Th17, and Tr1/exTh17 cells. *Tec^−/−^* eYFP^+^ cells from small intestine or colon of diseased mice showed a high degree of plasticity of Th17 cells towards Th1/exTh17 cells, as *Tec^−/−^* eYFP^+^ had a higher frequency of eYFP^+^ IFN-γ^+^IL-17A^–^ cells in comparison with WT cells (**Figures 5C, D**; **Supplementary Figures S9D, E**). In addition, these *Tec^−/−^* Th1/exTh17 cells showed enhanced IFN-γ production, as MFI of these cells was significantly higher compared with WT Th1/exTh17 cells in small intestine (**Figure 5E**) and as a tendency in colon (**Supplementary Figure S9F**). However, IL-10^+^ cell frequencies were not increased among YFP^+^ intestinal cells in the absence of Tec (**Supplementary Figures S10B, C**). Taken together, these data indicate that Tec negatively regulates Th17 cells in the gut during onset of disease, restraining their plasticity towards Th1/exTh17 cells.

### Tec Regulates IL-23R Expression in eYFP^+^ Cells in Small Intestine and Colon During Onset of Colitis

One of the most important molecular factors regulating Th17 plasticity is IL-23 (50), which was shown to promote expression of IFN-γ in Th17 cells and their transdifferentiation towards Th1 cells (51). The IL-23R has to be upregulated on Th17 cells during their differentiation in response to IL-6 (16). Thus, mechanisms that enhance IL-23R levels on Th17 cells also promote their plasticity. We therefore monitored the expression of the IL-23R on Th17, IFN-γ^+^Th17, and Th1/exTh17 cells from small intestine or colon of diseased mice. We observed a significant higher frequency of IL-23R-positive cells among *Tec^−/−^* Th17, IFN-γ^+^Th17, and Th1/exTh17 cells from small intestine (**Figures 5F, G**, upper panel) correlating with higher IL-23R expression levels (**Figure 5G**, lower panel). The frequency of IL-23R-positive cells was enhanced among *Tec^−/−^* IFN-γ^+^Th17 cells from colon origin (**Supplementary Figures S9G, H**, upper panel) correlating with a tendency towards higher receptor levels (**Supplementary Figure S9H**, lower panel). Taken together, our data show enhanced IL-23R-positive cells with higher receptor levels among eYFP^+^ cells in the intestine in the absence of Tec during onset of colitis.

### Loss of Tec in CD4^+^ T Cells Leads to an Enhanced Disease Severity During T-Cell-Driven Colitis

**Figure 6:**
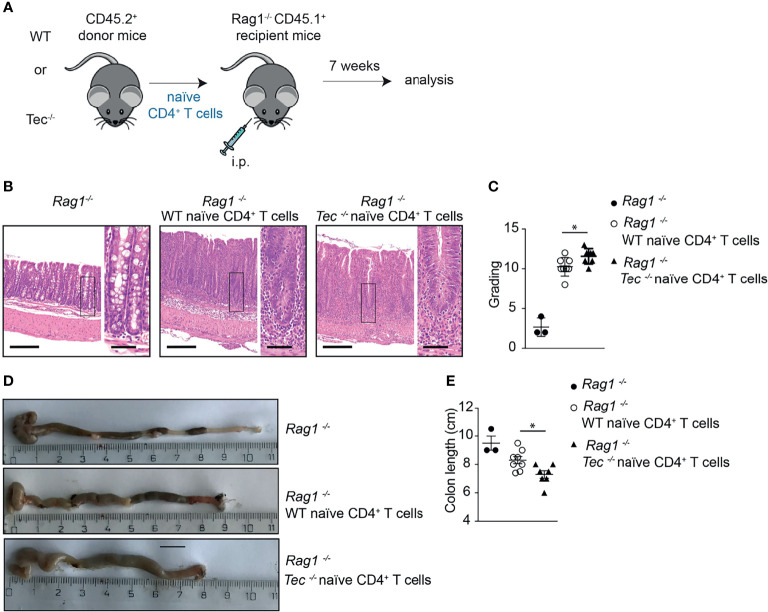
Tec impacts on disease severity during overt T cell-driven colitis. **(A)** Experimental scheme. **(B)** Representative histological images of colon from WT healthy control, and WT and *Tec-/-* diseased mice. HE-staining was performed. Bars represent 200µm and 50µm, respectively. **(C)** Disease score as calculated by adding scores from assessment of cell infiltrate, epithelial changes and lesions of the mucosal architecture as described in *Materials and Methods*. **(D)** Representative picture of colon of WT healthy control, and WT and *Tec-/-* diseased mice. **(E)** Summary of **(D)**. **(C, E)** show the summary of three experiments with at least two animals per group. *P* values were calculated by an unpaired Two-tailed t test. *P < 0.05.

To understand whether the altered Th17 differentiation in the absence of Tec also affects the disease severity in the T-cell-driven colitis model, a histopathological analysis of the colon from *Rag1^−/−^* mice 7 weeks after transfer of WT or *Tec^−/−^* naïve CD4^+^ T cell was performed (**Figure 6A**). At this time point, disease severity is pronounced (44). In general, colitis was significantly induced irrespective whether WT or *Tec^−/−^* naïve CD4^+^ T cell were transferred as revealed by transmural infiltration with immune cells, epithelial cell hyperplasia, loss of goblet cells, crypt abscesses, and focal erosions (**Figure 6B**). A score was assigned by assessing all these parameters and revealed a moderate but significantly stronger inflammation in colons of animals that received *Tec^−/−^* naïve CD4^+^ T cells in comparison with those receiving WT naïve CD4^+^ T cells (**Figure 6C**). In agreement, colon length was significantly shorter in mice that received *Tec^−/−^* naïve CD4^+^ T cells, further indicating that a stronger colon inflammation was induced by *Tec^−/−^* naïve CD4^+^ T cells (**Figures 6D, E** **)**. These data together support an increased pathogenicity of Th17 subsets generated during colitis in the absence of Tec, either because of increased frequency of Th17 cells or because of the occurrence of more pathogenic-type Th17 cells, or both.

### Tec Regulates Th17 Frequency and Plasticity During Inflammatory Stage of Colitis

**Figure 7:**
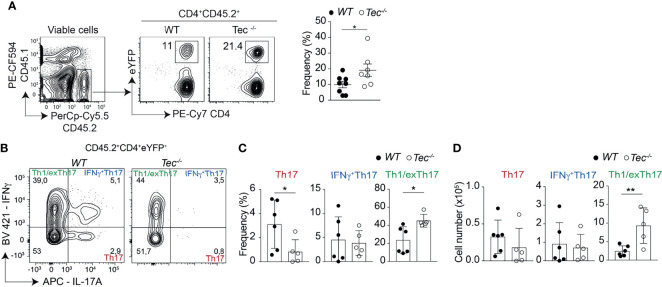
Tec supports Th1/exTh17 cells during overt T cell-driven colitis. **(A)** Dot plots represent the frequency of eYFP^+^ CD4^+^ T cells from LPL after gating on transferred CD45.2^+^ cells. The summary of three experiments with at least two animals per group is represented alongside. **(B)** Dot plots represent the frequency of IL-17A and IFNγ positive LPLs gated on eYFP^+^ CD4^+^ T cells from WT and *Tec-/-* diseased mice. **(C)** Summary of **(B)**. **(D)** Summary of total cell numbers of IL-17A and IFNγ single and double producing cells. **(C, D)** show the summary of three experiments with at least two animals per group. P values were calculated by an unpaired Two-tailed t test. *P < 0.05, **P < 0.01.

To assess whether Th17 cell differentiation and plasticity is also altered during an inflammatory stage of colitis where disease is pronounced, we analyzed intraepithelial lymphocytes (IEL) and lamina propria lymphocyte (LPL) subsets 7 weeks after transfer. We observed more eYFP^+^ cells among small intestine associated IEL and LPL of diseased *Tec^−/−^* mice compared with diseased WT mice (**Figure 7A**; **Supplementary Figure S11**). In addition. Th1/exTh17 LPLs were significantly increased in frequency and number in the absence of Tec (**Figures 7B–D**; **Supplementary Figure S12A** for a gating strategy). IL-10 production was not changed between eYFP^+^ WT and *Tec^−/−^* LPLs (**Supplementary Figures S12B–D** **)**. Th1 cells (eYFP^−^) that did not originate from Th17 cells were strongly induced in both experimental groups, however no difference was observed in the frequency between WT and *Tec^−/−^* Th1 cells (**Supplementary Figures S12E, F**). Taken together, Tec-deficient naïve CD4^+^ T cells differentiated markedly more towards effector Th17 cells with enhanced plasticity towards Th1/exTh17 cells during overt colitis with pronounced inflammatory phenotype.

## Discussion

In this study, we examined the role of the tyrosine kinase Tec in Th17 cell differentiation and during T-cell- driven colitis, a Th1/Th17-driven disease. As Th17 cells play a major role in autoimmune or inflammatory diseases, it is of key importance to identify signaling networks regulating their differentiation and function. We found that Tec isoform a was predominantly induced by the Th17 driving cytokines TGF-β1 and IL-6, implying a specific function of this kinase during Th17 differentiation. We further demonstrate that Tec fine tunes the response to IL-6 *via* STAT3, counteracting the upregulation of the *Il23r*. Moreover, Tec negatively regulated Th17 differentiation and pathogenicity during immunization and colitis *in vivo* in a T-cell intrinsic way.

Systems biology studies (24) showed by computational analysis that Tec is upregulated in Th17 cells, located in a Th17-specific signal transduction node and being under control of the transcription factor RORγt. It was however not known which Th17-driving conditions led to its upregulation and what implication this upregulation has on the Th17 differentiation itself and the subsequent immune response. An important finding of this study is the cytokine-inducible nature of Tec expression, being under control of TGF-β1 and reaching even higher levels by further addition of IL-6. Other cytokines important for Th17 differentiation and expansion such as IL-23 or IL-1β did not affect the levels of Tec. We therefore determined two crucial cytokines which are required for Th17 differentiation and also regulate Tec expression.

It was previously reported that Tec is expressed at very low levels in naïve T cells, being upregulated upon activation, and in particular in Th2 cells (36). We did not observe a significant upregulation of Tec in activated T cells in the absence of exogenous cytokines in contrast to previous publications (36). The discrepancy might be due to the purification of the CD4^+^ T cells, as our cells are naïve cells sorted based on CD44 and CD62L markers and do not contain residual effector or memory like T cells which display a higher Tec kinase expression compared with naïve T cells and expand during activation (28). Stimuli/cytokines other than TGF-β1 and IL-6 might induce Tec upregulation after T-cell activation, as Tec was shown to be upregulated in Th2 cells, in contrast to Th1 cells. In previous studies, we did however not observe an alteration in Th2-cell differentiation in the absence of Tec and Tec-deficient mice displayed no change in disease severity or outcome in a model of Th2-driven allergic airway inflammation (28). As IL-6 signaling is also important for Th2- but not Th1-cell differentiation (52), we cannot exclude a function of Tec in the IL-6 sensing during Th2 differentiation, with an impact on other Th2-driven diseases such as worm infections. Further studies aiming at deciphering the exact cytokine condition required for Tec upregulation during Th2 differentiation might uncover such a mechanism.

In addition to Jak proteins, Tec has been shown to be associated with gp130, and a positive function in signal transduction from the IL-6 receptor (IL-6R) was hypothesized for Tec (53). Our data in contrast define Tec as a fine-tuning negative regulator of the IL-6 signaling pathway. The Src homology region 2 domain-containing tyrosine phosphatase-1 (SHP-1) is a cytoplasmic protein tyrosine phosphatase expressed in all hematopoietic cell lineages which was shown to dampen IL-6 signaling and restrain Th17 differentiation (54). It is therefore tempting to speculate that Tec promotes recruitment of SHP-1 to the IL-6R signaling complex, thereby mediating its inhibitory effects. Another likely molecular mechanism is suggested by the kinetic study of STAT phosphorylation. At low IL-6 concentration, we observed in absence of Tec an increase in STAT1 (although only a tendency) and STAT5 phosphorylation and the strongest effect on STAT3 phosphorylation 48 hours after the cultures were started. This kinetic correlates with the regulation of SOCS3 expression on protein level (55) and its inhibitory function on STAT proteins (56). As SOCS3 was shown to interact with Tec (37, 57), Tec might play a function in the SOCS3-mediated inhibition of STAT proteins downstream of the IL-6R. Further studies deciphering the precise molecular interaction of Tec with SHP-1, SOCS3, and other known negative regulators of Th17-cell differentiation or IL-6R signaling such as SOCS1 (58), SHP-2 (59), or TNFR-associated factors 2 and 5 (TRAF2/5) (60) are warranted.

Another important finding of our study is that Tec not only dampens Th17 differentiation *in vitro* but also *in vivo* in a T-cell intrinsic way. We analyzed Th17 cell plasticity based on the IL-17A fate mapping mouse. As not all Th17 cells are marked after expressing IL-17A due to Cre protein levels, not all Th17 cell fates can be traced. Therefore, some transdifferentiation mechanisms might not be possible to analyze due to this technical consideration and will be followed up by using for instance IL-17A reporter mice and transfer systems. However, the use of IL-17A fate mapping mice combined with *in vivo* models allowed us to draw conclusions as to Th17 plasticity towards the Th1 subset in absence of Tec. By using a short-term immunization approach over 6 days, we identified more IL-17A and IFN-γ coproducing Th17 cells, while in a long-term T-cell-driven colitis model over 7 weeks we observed more Th1/exTh17 cells in the absence of Tec. The difference in the type of Th17 subset that is enhanced in knockout cells in these two different *in vivo* models could be related to the difference in the analyzed *in vivo* sites and different cytokine environments to which *Tec^−/−^* naïve CD4^+^ T cells were exposed, or it could indicate that the plasticity of Th17 cells towards Th1/exTh17 cells occurs gradually over time, and that IFN-γ^+^Th17 cells are an intermediate subset derived from Th17 cells which ultimately lose their IL-17A secretion capacity. In the latter hypothesis, our data identify Tec as an important factor which restrains this process. In addition, disease severity was increased in the T-cell-driven colitis model when Tec-deficient cells were transferred, implying also a higher pathogenic potential of Th17 cells in absence of Tec, either because of the increase in Th17 frequency, or because of the increased frequency of Th1/exTh17 cells with higher pathogenic potential, or both. Our data together show the important function of Tec in restraining Th17 differentiation, pathogenicity and plasticity.

*In vitro*, low IL-6 concentration led to a higher *Il23r* expression on Tec-deficient Th17 cells. IL-23 was shown to be a crucial factor for the induction of Th17 pathogenicity and plasticity, strongly promoting the generation of IFN-γ^+^Th17 or Th1/exTh17 cells (51). In addition, IL-23 is essential for the emergence of IFN-γ^+^Th17 during colitis (61). It is therefore tempting to speculate that, in the absence of Tec, naïve CD4^+^ T cells which encounter low IL-6 concentrations *in vivo* differentiate more robustly towards IFN-γ^+^Th17 and Th1/exTh17 cells as a result of higher IL-23R induction and therefore also a stronger susceptibility to IL-23. In favor of this hypothesis is the higher IL-23R expression *in vivo* in *Tec^−/−^* Th17, IFN-γ^+^Th17, and Th1/exTh17 cells from small intestine of animals with colitis. Therefore, *in vivo*, Tec might regulate the IL-6R signaling pathway and/or additional signaling pathways and thereby lead to an attenuation of Th17 differentiation and concomitant plasticity, restraining in the same process Th17 pathogenicity.

A limitation to our study is that Tec was deleted in all T cells and not specifically in Th17 cells. Therefore, we cannot exclude the possibility that T-cell subsets different from Th17 cells and regulated by Tec impact on Th17 plasticity indirectly. Studies based on conditional knockout mice are warranted to fully address this issue. However, as the IL-23R is upregulated in Th17 cells in the T-cell-driven colitis model in the absence of Tec, it is tempting to speculate that Tec kinase regulates Th17 plasticity in an Th17-intrinsic mode by acting in the IL-6R signaling pathway.

Taken all our data together, we propose the following main working model and mechanism. During T-cell activation and after exposure to TGF-β1 and IL-6, Tec is upregulated and acts as negative feedback loop on IL-6R signaling. This is comparable with the SOCS proteins which are induced by IL-6R signaling and function as a negative feedback control (62). The further requirement of TGF-β1 signaling for Tec induction adds a level of control and specificity for Th17-cell differentiation. After Tec induction under high IL-6 concentrations, the positive regulators like Jak and STAT3 might outnumber negative factors such as Tec and a threshold STAT3 phosphorylation and translocation can be achieved, allowing optimal Th17 differentiation (**Supplementary Figure S13A**, left panel). At low IL-6 concentrations, the positive regulators are counteracted by the inhibition of Tec (**Supplementary Figure S13A**, middle panel). This leads to lower IL-23R levels at the surface of the cells, and lower susceptibility to differentiate towards pathogenic Th17 cells with increased plasticity. In absence of Tec, the IL-6R signaling at low IL-6 concentrations is not inhibited by Tec, and results in higher STAT3 phosphorylation and translocation, higher Th17 differentiation and IL-23R levels on the cell surface (**Supplementary Figure S13A**, right panel). Following the tunable diffusion-consumption mechanism of cytokine propagation proposed by Oyler-Yaniv et al. (63) which was shown to support cell plasticity, we propose a model *in vivo* where an IL-6 producing cell surrounded by activated CD4^+^ T cells is the center of a cytokine niche with a defined size (**Supplementary Figure S13B**). In inflammatory conditions and in particular even more at mucosal sites CD4^+^ T cells are exposed to IL-23 (61). CD4^+^ T cells in close proximity of the IL-6 producing cell will have a high IL-6R signaling, and therefore strongly upregulate IL-23R and differentiate to pathogenic Th17 cells that might even transdifferentiate to Th1 cells. CD4^+^ T cells that locate further away from the IL-6-producing cell will receive less cytokine signal, and differentiate to Th17 cells with low to no pathogenicity and plasticity. In the absence of Tec however, the sensitivity of these cells to IL-6 is enhanced, and therefore cells at the border of the cytokine niche are receptive to IL-6 signals, leading to more Th17-cell differentiation, pathogenicity, and plasticity (**Supplementary Figure S13B**).

Our data show a more pronounced plasticity of Tec-deficient Th17 cells towards Th1 cells. In an *S. pneumoniae* immunization and lung infection model, more *Il17a* was expressed in lungs in the absence of Tec accompanied by a reduced bacterial burden without aggravation of tissue damage (28). One possible explanation of this positive disease outcome is that in absence of Tec, more IFN-γ^+^Th17 or Th1/exTh17 cells were generated which however in this context rather helped in controlling bacterial infection, therefore reducing bacterial spread and disease severity. Pathogen-specific Th17 cells expressing either IFN-γ or IL-10 have been described (64). Therefore, IFN-γ^+^Th17 or Th1/exTh17 cells might have a context-dependent detrimental or beneficial outcome. Follow-up studies based on vaccination and infection experiments are warranted to test whether Tec might be an interesting target for enhancing Th17-specific mucosal immunity against extracellular bacteria (53) and whether Tec-specific inhibitors might be considered as vaccine adjuvants.

In conclusion, our results provide another example how the expression of signaling molecules is regulated in a T helper subset-specific manner, allowing the immune system to fine tune the response against harmful pathogens while restraining inflammatory diseases. Therefore, Tec can be considered a gatekeeper preventing immune overreaction at low IL-6 levels.

## Data Availability Statement

The original contributions presented in the study are included in the article/**Supplementary Material**. Further inquiries can be directed to the corresponding author.

## Ethics Statement

The animal study was reviewed and approved by the Austrian Federal Ministry for Education, Science and Art.

## Author Contributions

LS, MA, NB and WE designed the research; LS, MA and NB performed most of the experiments and analyzed the data; CZ, AH, SH, RR, LG, OS and SS performed some of the experiments; NB wrote the manuscript and WE helped in the manuscript editing. All authors contributed to the article and approved the submitted version.

## Funding

This work was supported by the Austrian Science Fund (FWF) through the projects P24265, P30885, P27747 (P24265 and P30885 to NB and P27747 to SS), and FWF Special Research Program F70 (F07004 to NB and F07005 to WE). Further support was provided by the FWF and Medical University of Vienna doctoral program (DOC 32) “TissueHome” to WE.

## Conflict of Interest

The authors declare that the research was conducted in the absence of any commercial or financial relationships that could be construed as a potential conflict of interest.

## Publisher’s Note

All claims expressed in this article are solely those of the authors and do not necessarily represent those of their affiliated organizations, or those of the publisher, the editors and the reviewers. Any product that may be evaluated in this article, or claim that may be made by its manufacturer, is not guaranteed or endorsed by the publisher.

## Abbreviations

FBS, fetal bovine serum; MFI, mean fluorescence intensity; PBS, phosphate-buffered saline; PMA, phorbol 12-, mystriate 13-acetate; qRT-PCR, quantitative real-time PCR; Th17, T helper 17; WT, wild type; n.s., not significant.
