# Supplementary material for: The Tyrosine Kinase Tec Regulates Effector Th17 Differentiation, Pathogenicity, and Plasticity in T-Cell-Driven Intestinal Inflammation
Source: Front Immunol. 2021 Dec 21;12:750466. doi: 10.3389/fimmu.2021.750466 (PMC8728872; doi:10.3389/fimmu.2021.750466)
Supplement: Supplementary file 1 [file DataSheet_1.pdf]

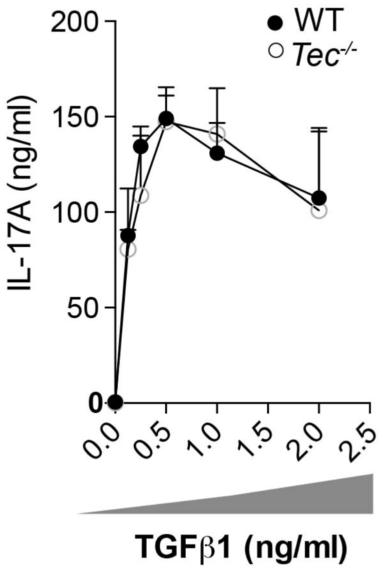

Supplementary Figure 1 Tec does not impact on TGFβ1 sensing of naïve CD4+ T cells during Th17 differentiation. IL-17A secretion was determined in the supernatant of cultures of naïve CD4+ T cells isolated from either *WT* or *Tec* -/- mice and stimulated with immobilized anti-CD3 and anti-CD28 for 3 days under increasing TGFβ1 concentrations and 20 ng/ml IL-6. The quantification of three experiments with three replicates per group is shown. P values were calculated by two-way ANOVA using a Sidak's multiple comparisons test.

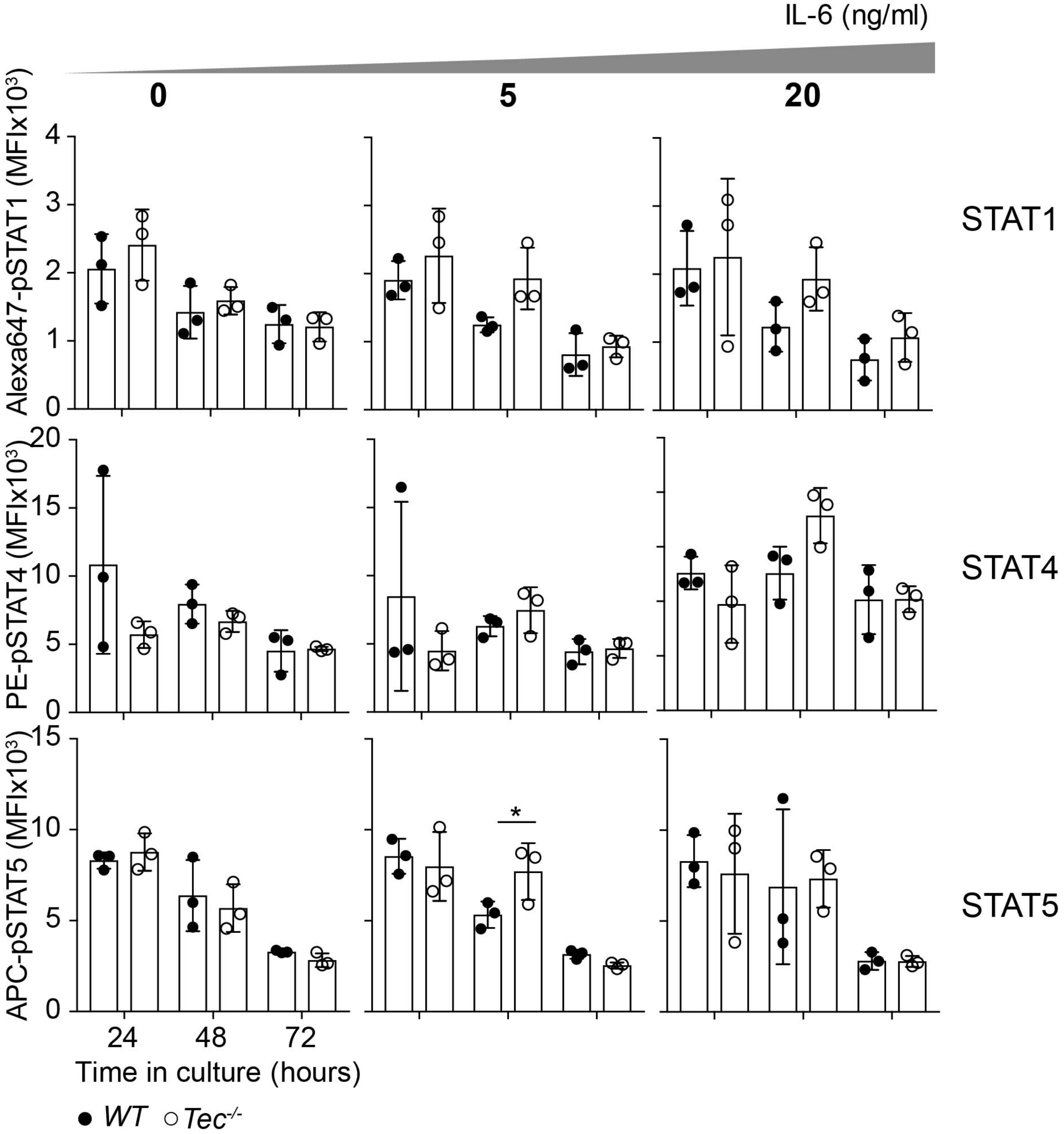

Supplementary Figure 2 Phosphorylation of STAT1, STAT4 and STAT5 in respnse to IL-6 in absence of Tec. Naïve CD4+ T cells from WT or *Tec*-/- mice were cultured with anti-CD3 and anti-CD28 for the indicated time points with 1 ng/ml TGFβ1 and in abscence or presence of 5 or 20 ng/ml IL-6. Phosphorylation of STAT1, STAT4 and STAT5 was measured by flow cytometry. The scatter plots are the summary of 3 independent experiments. P values were calculated by two-way ANOVA using a Sidak's multiple comparisons test *= P<0.05.

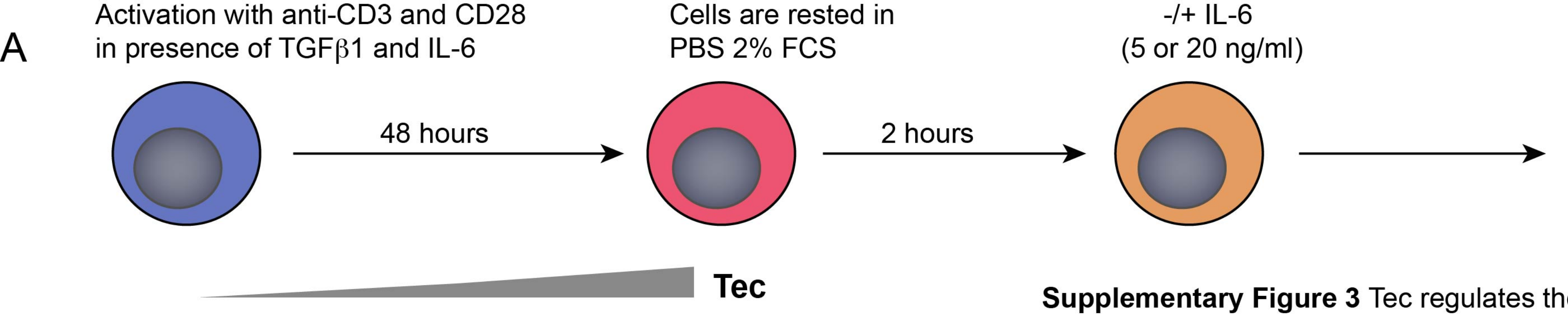

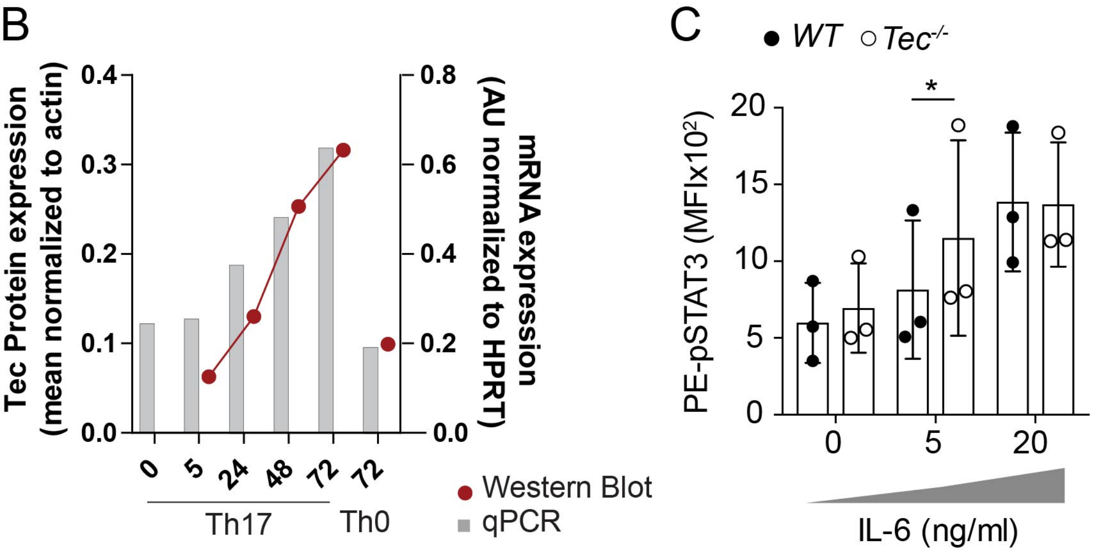

Supplementary Figure 3 Tec regulates the IL-6 receptor signaling directly. (A) Experimental scheme. (B) Naïve CD4⁺ T cells from WT or *Tec*$^{-/-}$ mice were cultured with anti-CD3 and anti-CD28 for the indicated time points with 1 ng/ml TGFβ1 and 20 ng/ml IL-6 (Th17), or no added cytokine (Th0). Tec protein expression was measured by immunoblotting and *Tec* mRNA expression by qPCR analysis. (C) WT and *Tec*$^{-/-}$ naïve CD4^{+} T cells were treated as in (A) and STAT3 phosphorylation was analyzed by flow cytometry. The scatter plot shows the summary of three independent experiments. P values were calculated by two-way ANOVA using a Sidak's multiple comparisons test. *= P<0.05.

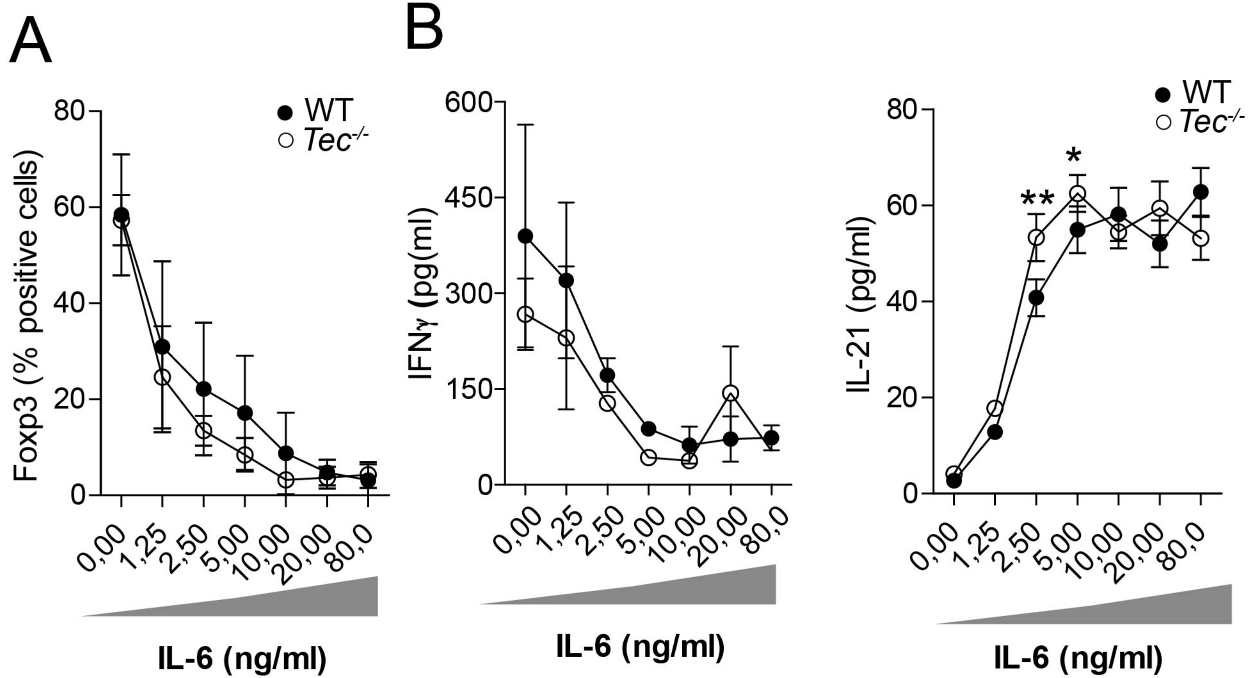

Supplementary Figure 4 Tec does not alter Th17 plasticity *in vitro.* (A) The frequency of Foxp3 positive cells were determined by flow cytometry among naïve $CD4^+$ T cells isolated from either *WT* or *Tec* $^{-/-}$ mice and stimulated with immobilized anti-CD3 and anti-CD28 for 3 days under increasing IL-6 concentrations and 1 ng/ml TGFβ1. The quantification of three experiments is shown. (B) IFNγ and IL-21 secretion was determined in the supernatant of cultures of naïve $CD4^+$ T cells isolated from either *WT* or *Tec* $^{-/-}$ mice and stimulated with immobilized anti-CD3 and anti-CD28 for 3 days under increasing TGFβ1 concentrations and 20 ng/ml IL-6. The quantification of three experiments is shown. P values were calculated by two-way ANOVA using a Sidak's multiple comparisons test. *= P<0.05, **=P<0.01.

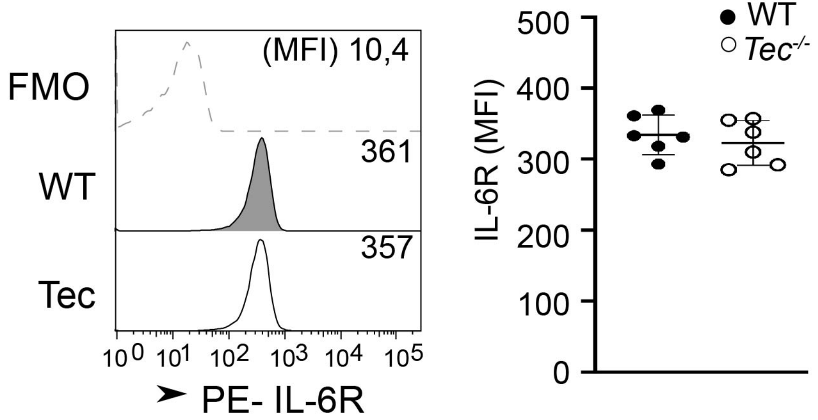

Supplementary Figure 5 IL-6R levels are not altered on naïve CD4^{+} T cells in absence of Tec. Flow cytometric analysis of IL-6Rα expression in live single CD44^{lo}CD62L^{+}CD4^{+}TCRβ^{+}CD19^{-} splenocytes isolated from *WT* or *Tec* $^{-/-}$ mice. One representative experiment out of two is shown. FMO, Fluorescence Minus One. MFI, Mean Fluorescence Intensity.

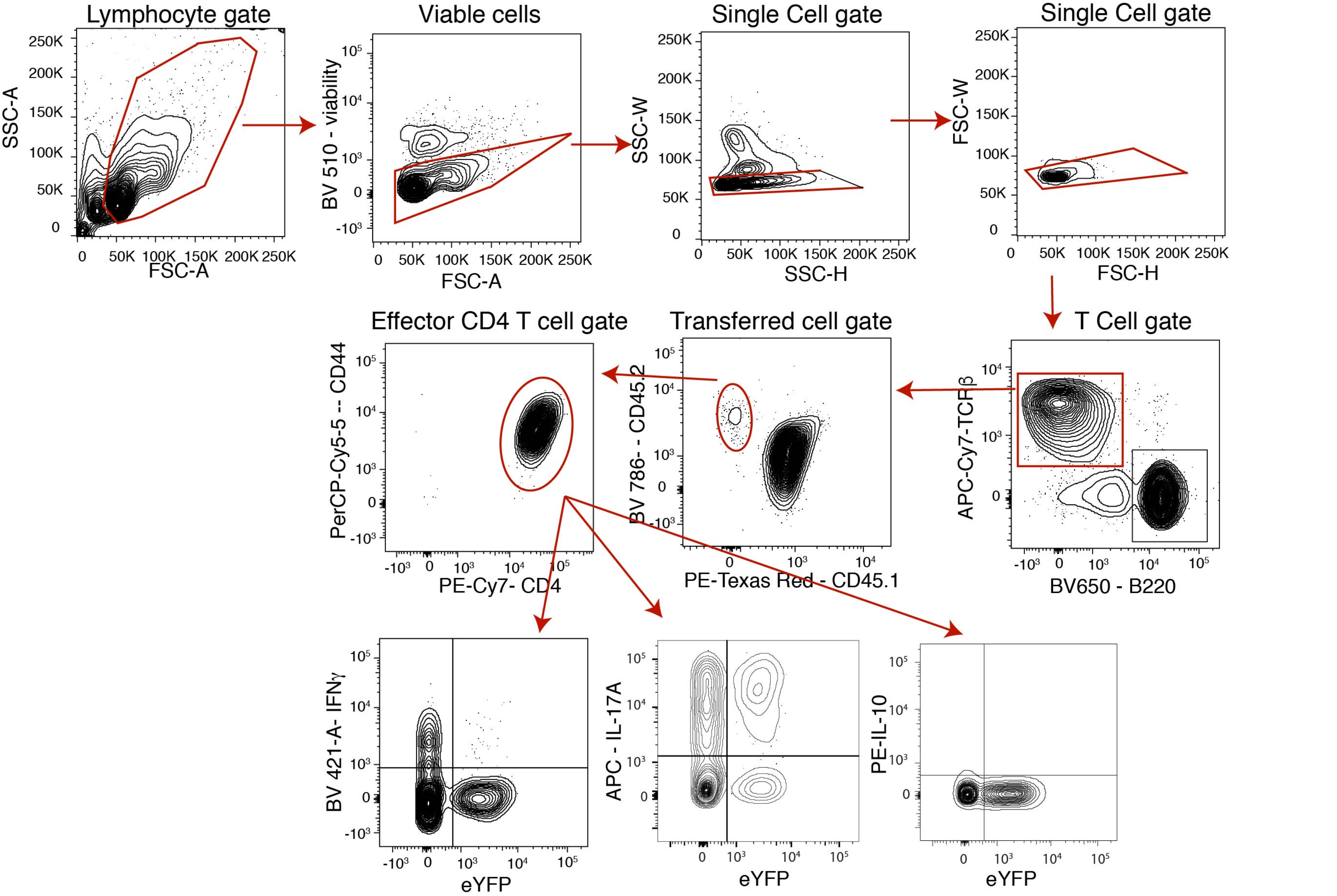

Supplementary Figure 6 Gating strategy for analysis of Th17 cells in draining lymph nodes 6 days after transfer of *WT* or *Tec* $^{-/-}$ naïve OT-II IL-17A^{cre} R26^{YFP}

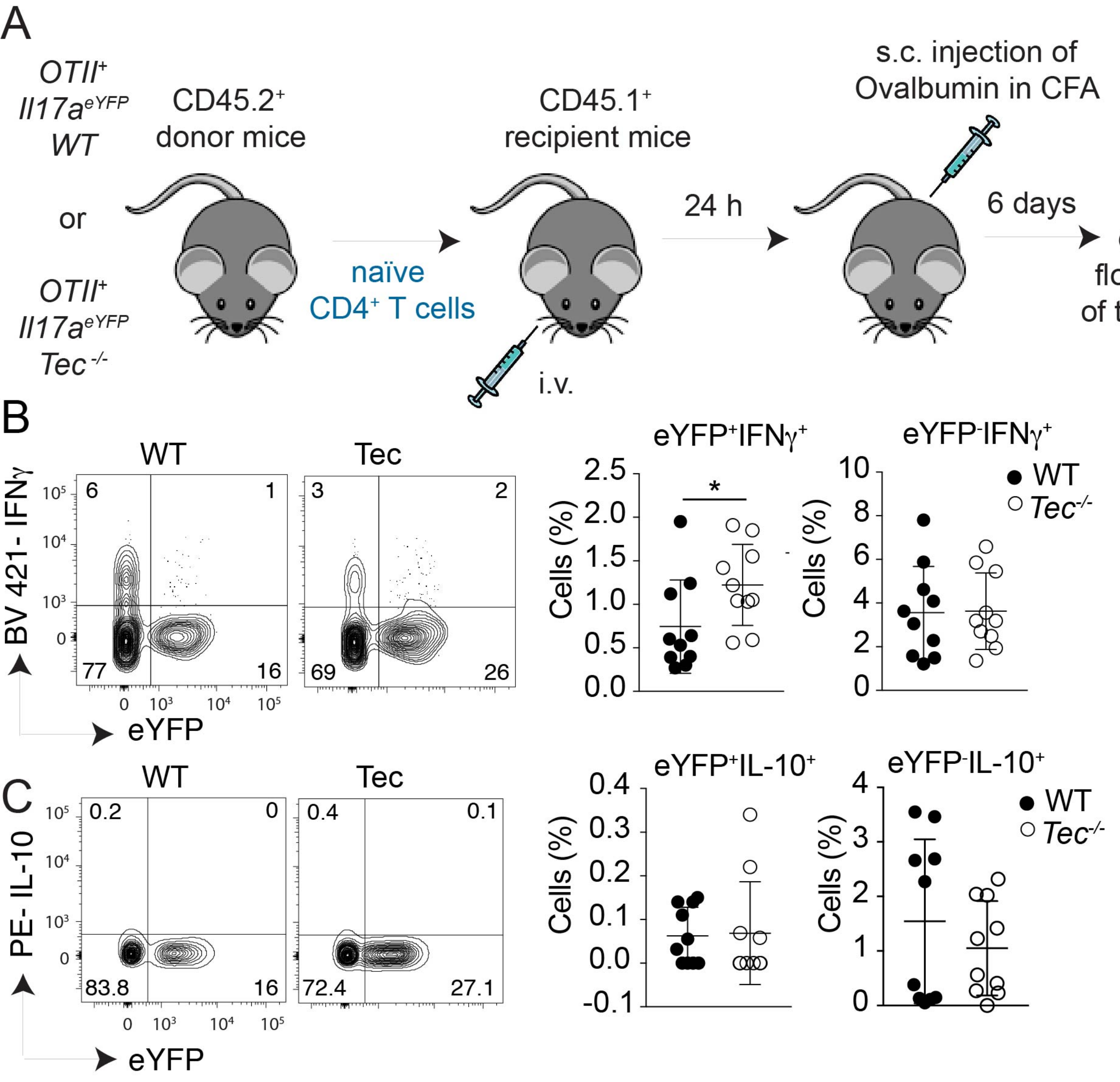

Supplementary Figure 7 Differentiation of T helper cells *in vivo* during immunization in absence of Tec. (A) Experimental scheme. (B) Frequency of IFNγ+ and eYFP+ CD4+CD45.2+ live T cells from *WT* and *Tec -/-* mice treated as in (A) (C) Frequency of IL-10+ and eYFP+ CD4+CD45.2+ live T cells from *WT* and *Tec -/-* mice treated as in (A) P values were calculated by an unpaired two-tailed t test. *= P<0.05.

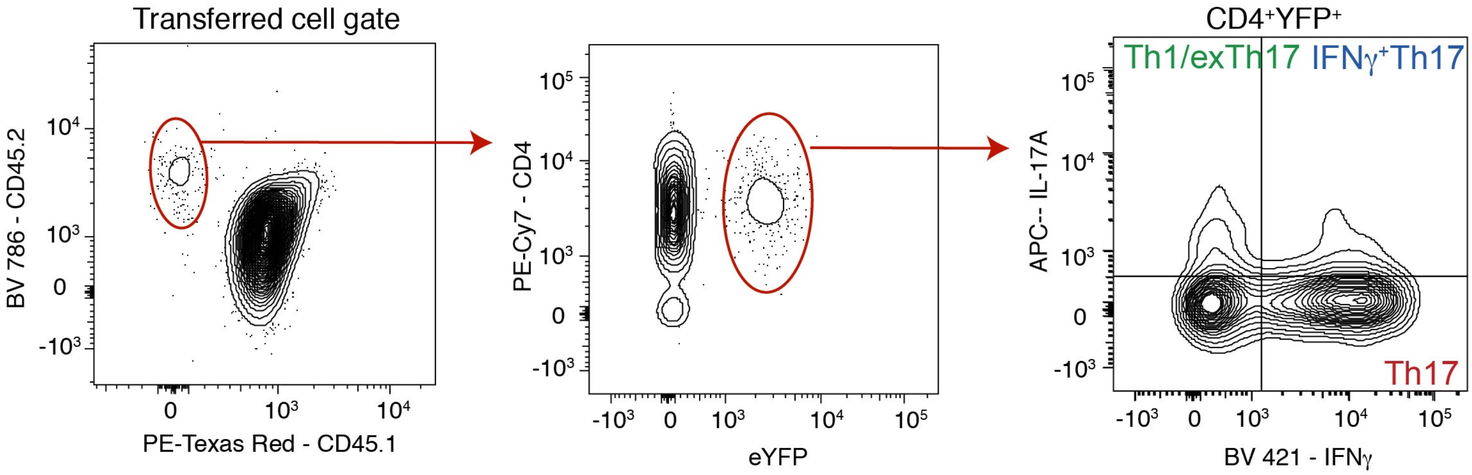

Supplementary Figure 8 Gating strategy for Figure 4.

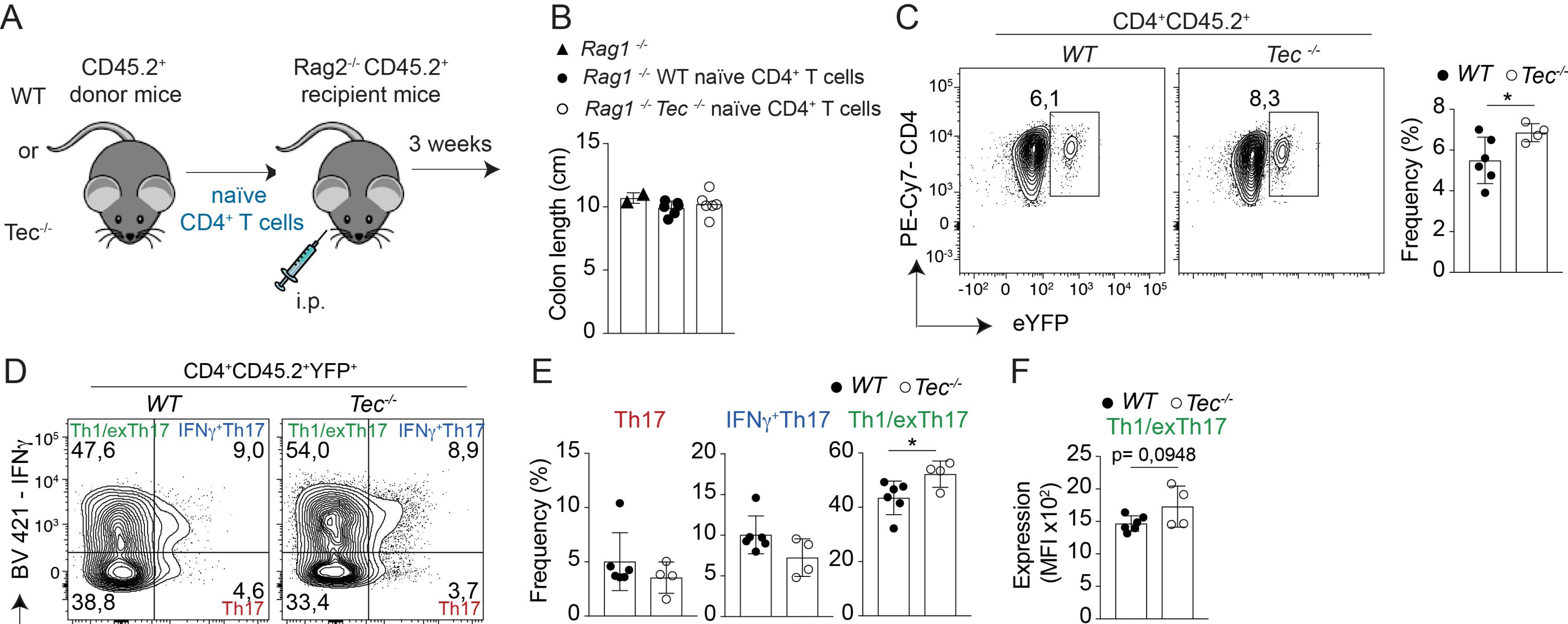

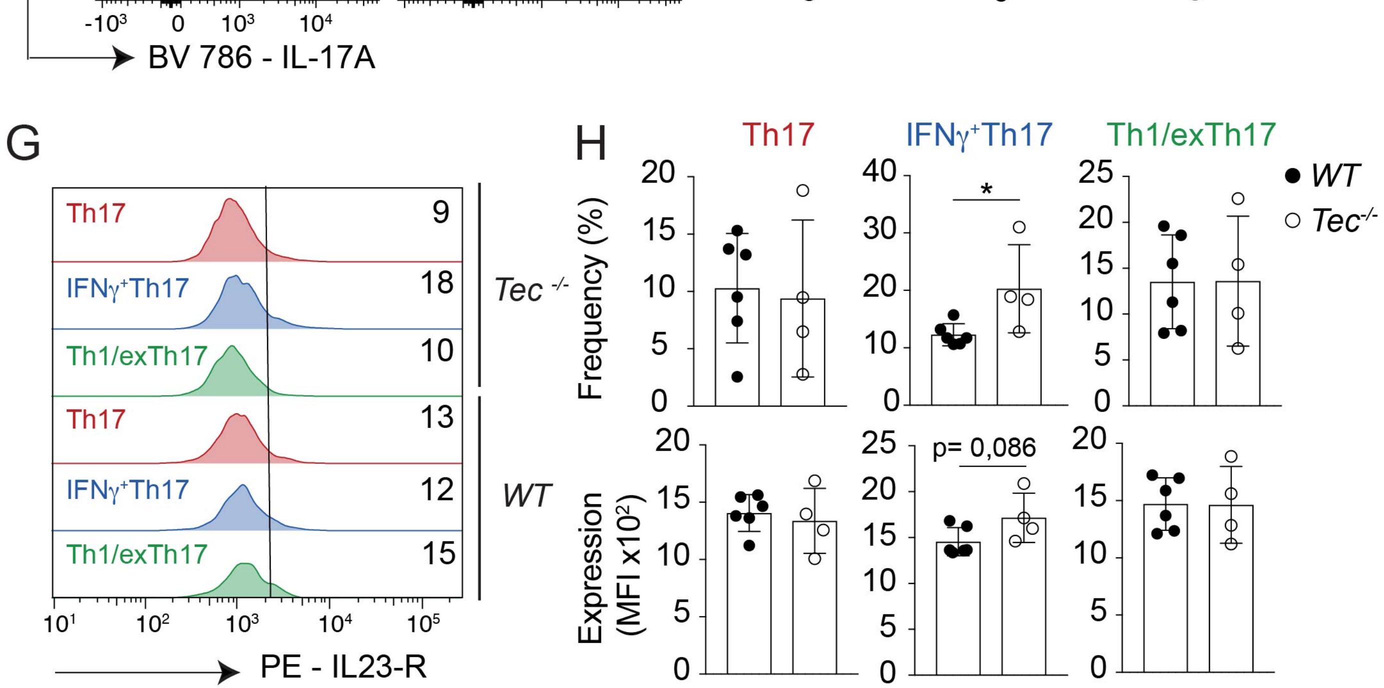

Supplementary Figure 9 Tec enhances Th17 differentiation and plasticity in the colon during onset of adoptive transfer colitis. (A) Experimental scheme. (B) Colon length of *Rag2*-/- mice with the indicated transferred cells. (C) Frequency of eYFP+cells from colon after gating on transferred CD4+CD45.2+ T cells. The summary is represented alongside. (D) Frequency of IL-17A+ and/or IFNγ+ i cells gated on eYFP+CD45.2+CD4+ T cells from WT and *Tec*-/- mice treated as shown in (A). (E) Summary of (D) (F) Expression level of IFNγ among CD45.2+CD4+eYFP+IFNγ+IL-17A- T cells gated as in (D). (G) IL-23R expression on the indicated cell type and genotype. The numbers represent the frequency of positive cells. (H) Summary of the frequencies (upper scatter plots) and expression levels (lower scatter plots) of the IL-23 R from cells represented in (F). (C,E, F and H) summarize two independent biological replicates with 3 animals per replicate for the WT group and 2 animals per replicate for the *Tec*-/- group. P values were calculated by an unpaired Two-tailed t test. *= P<0.05.

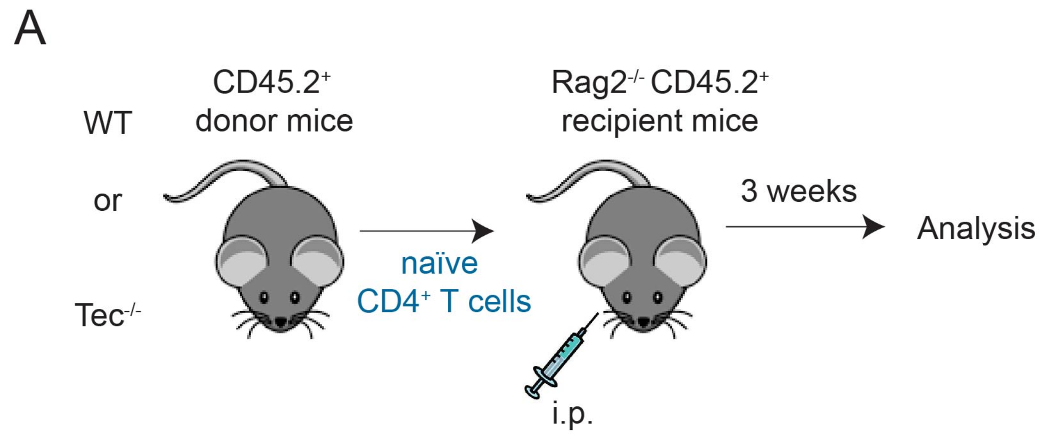

Supplementary Figure 10 Plasticity of Th17 cells towards IL10⁺Th17 or Tr1exth17 is not affected by Tec during onset of adoptive transfer colitis. (A) Experimental scheme. (B) and (C) Contour plot shows frequency of IL-17A and IL-10 single and double positive cells among WT and *Tec*$^{-/-}$ eYFP⁺ cells isolated from small intestine (B) and colon (C) of diseased *RAG2*$^{-/-}$ mice treated as in (A). Summary is shown alongside. (B) and (C) represent two independent biological replicates with 3 animals per replicate for the WT group and 2 animals per replicate for the *Tec*$^{-/-}$ group P values were calculated by an unpaired Two-tailed t test.

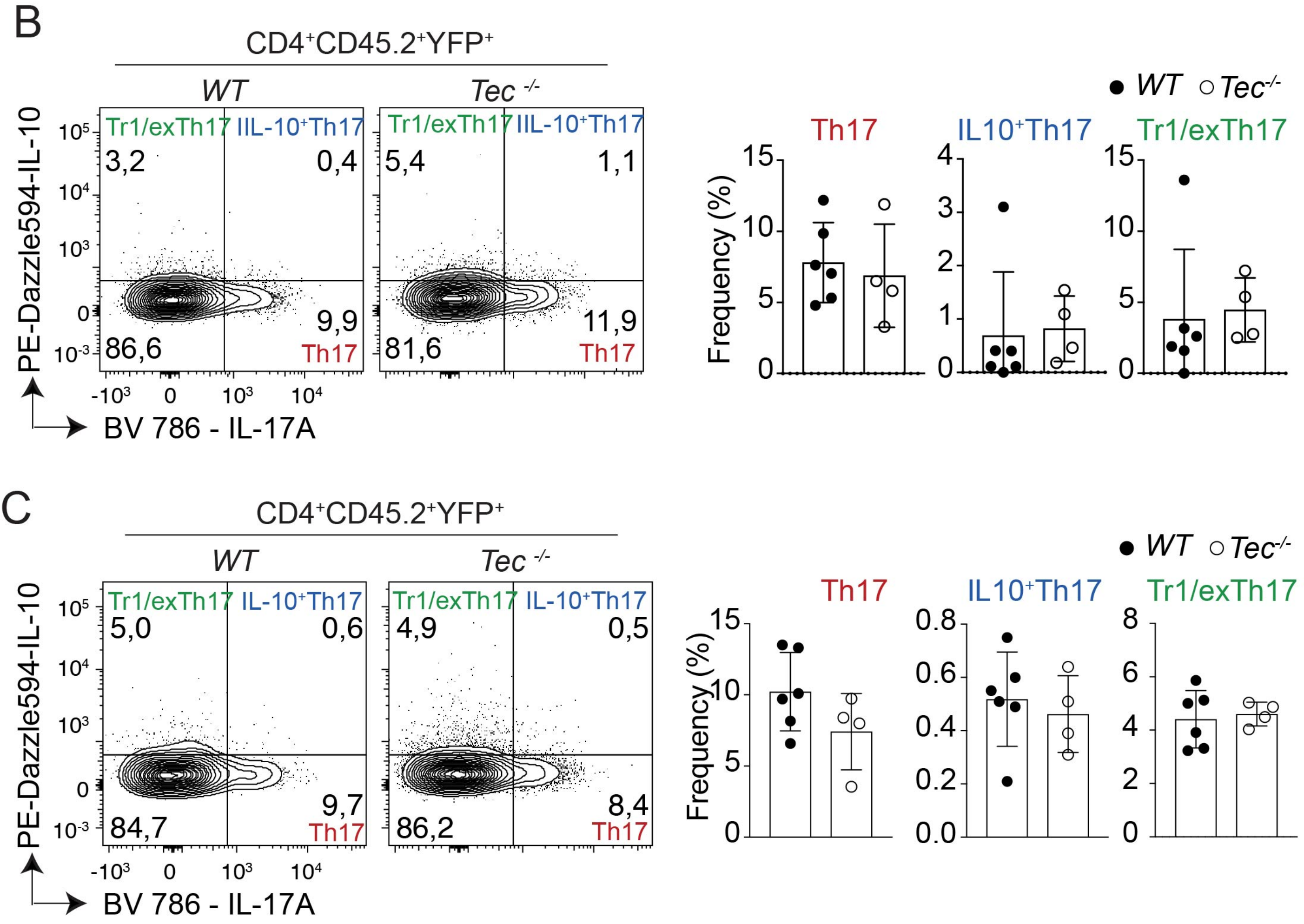

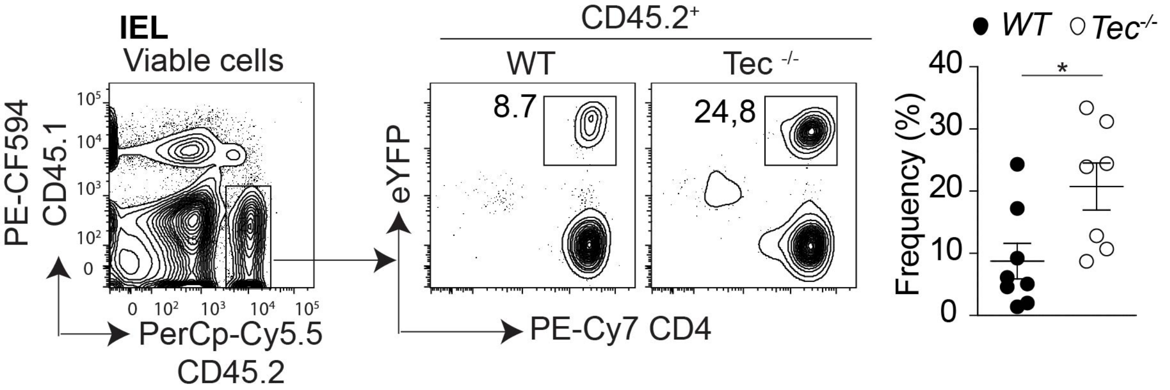

Supplementary Figure 11 Enhanced eYFP$^+$ cells in absence of Tec among IEL during overt adoptive transfer colitis. Contour plot represent the frequency of eYFP$^+$ cells after gating on CD45.2$^+$CD4$^+$ cells. A summary is shown alongside. P values were calculated by an unpaired Two-tailed t test. *= $P<0.05$.

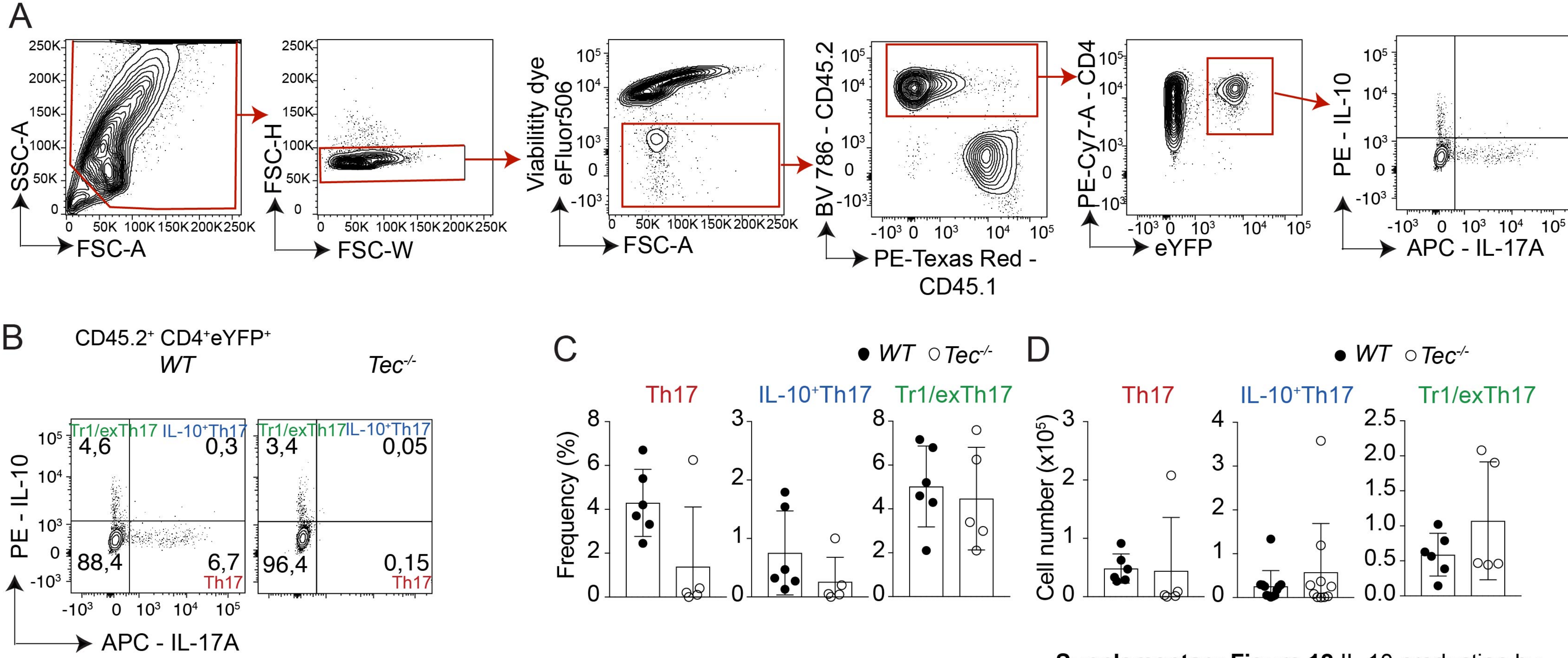

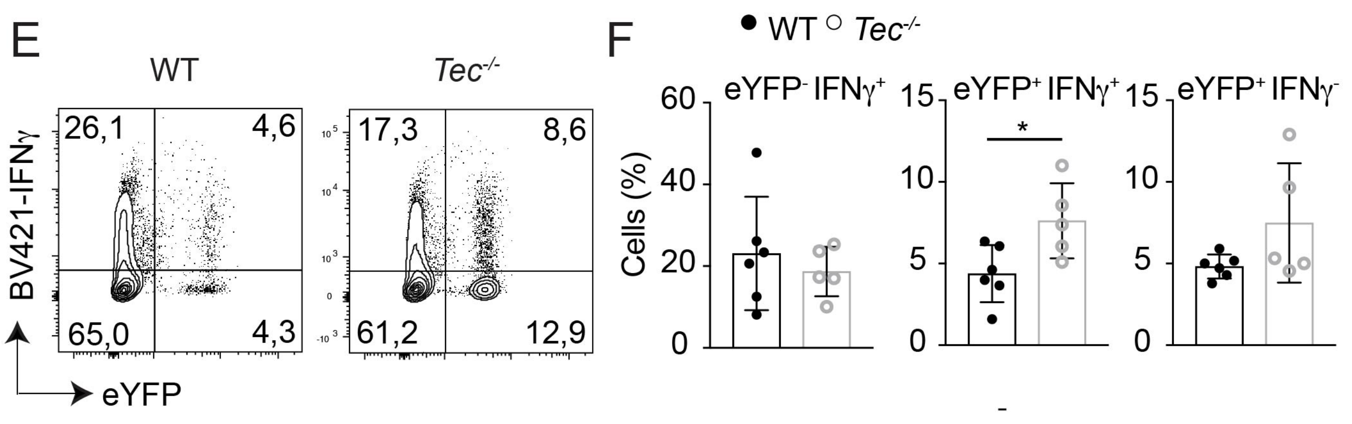

Supplementary Figure 12 IL-10 production by eYFP⁺ and eYFP⁻Th1 cells during colitis are not altered in the absence of Tec. (A) Overall gating strategy. (B) Dot plots represent the frequency of IL-17A and IL-10 positive LPLs gated on eYFP⁺ CD4⁺ T cells from WT and *Tec*-/- diseased mice. (C) Summary of (B). (D) Summary of total cell numbers of IL-17A and IL-10 single and double producing cells (E) Frequency of eYFP⁺ and IFNγ⁺ LPLs from *WT* and *Tec* -/- diseased mice. (F) Summary of (E) (B), (C), (E) and (F) show the summary of three experiments with at least two animals per group. P values were calculated by an unpaired Two-tailed t test. *= P<0.05

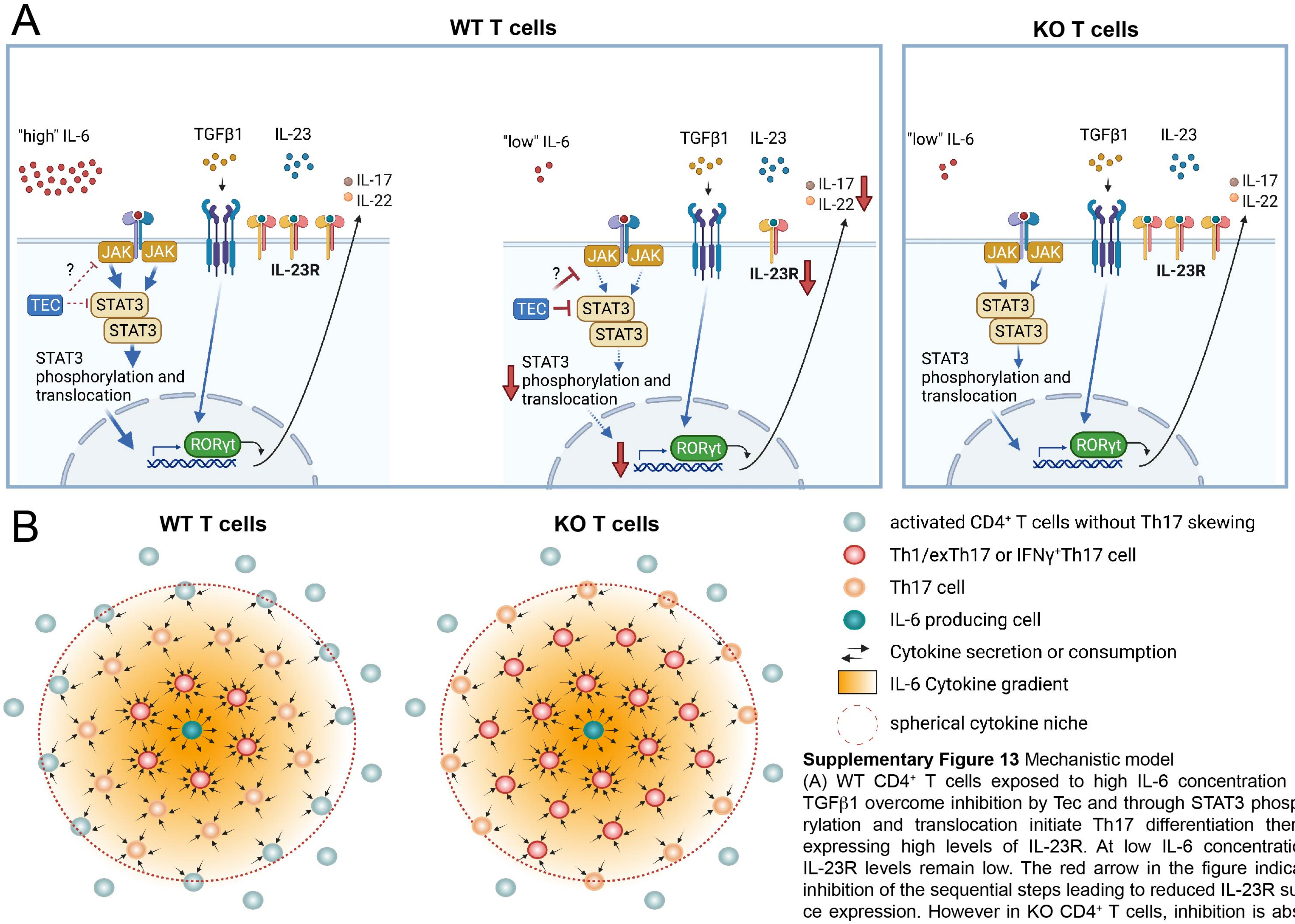

Supplementary Figure 13 Mechanistic model

(A) WT CD4⁺ T cells exposed to high IL-6 concentration and TGFβ1 overcome inhibition by Tec and through STAT3 phosphorylation and translocation initiate Th17 differentiation thereby expressing high levels of IL-23R. At low IL-6 concentrations, IL-23R levels remain low. The red arrow in the figure indicates inhibition of the sequential steps leading to reduced IL-23R surface expression. However in KO CD4⁺ T cells, inhibition is absent and at low IL-6 concentrations, IL-23R is strongly up-regulated.

(B) This model was adapted from Oyler-Yaniv, PMID: 28389069. An Il-6 producing cell is at the center of activated CD4⁺ T cells. CD4⁺ T cells in close proximity of the IL-6-producing cell get exposed to high IL-6 concentration, more distant ones get exposed to low IL-6 concentrations. Thus the closest cells to the IL-6 producer differentiate to Th17 cells with plastic potential, given their high expression of IL-23R. The distant CD4⁺ T cells differentiate to rather non-plastic Th17 cells, given their low expression of IL-23R. CD4⁺ T cells at the border of the cytokine niche don't differentiate to Th17 cells due to threshold IL-6 levels. In KO CD4⁺ T cells, more distant cells are more sensitive to IL-6 and strongly respond to low IL-6 concentrations, differentiating to Th17 cells with high plasticity. KO CD4⁺ T cells at the border of the spherical cytokine niche differentiate to Th17 cells due to their increased cytokine sensing. The figures were created with BioRender.
