# Supplementary material for: The Tyrosine Kinase Tec Regulates Effector Th17 Differentiation, Pathogenicity, and Plasticity in T-Cell-Driven Intestinal Inflammation
Source: Front Immunol. 2021 Dec 21;12:750466. doi: 10.3389/fimmu.2021.750466 (PMC8728872; doi:10.3389/fimmu.2021.750466)
Supplement: Supplementary file 2 [file Table_1.docx]

**Supplementary table** List of primers used in this study

| Primer | Sequence | Method | Reference |
| --- | --- | --- | --- |
| Tec sense | GGTTGGAGTGGTGAGGCTT | Real time | Felices et al., 2008 |
| Tec antisense | GGTAACGATGTAGATGGGC | Real time | Felices et al., 2008 |
| Tec iso fw | ATCCGTGTTGAGCTACTATGAGG | Isoform PCR |  |
| Tec iso rev | GTTCTGAGGAGCTGTTCTGCT | Isoform PCR |  |
